# Eye irritation testing of nanomaterials using the EpiOcular™ eye irritation test and the bovine corneal opacity and permeability assay

**DOI:** 10.1186/s12989-016-0128-6

**Published:** 2016-04-15

**Authors:** Susanne N. Kolle, Ursula G. Sauer, Maria C. Rey Moreno, Wera Teubner, Wendel Wohlleben, Robert Landsiedel

**Affiliations:** 1 BASF SE, Experimental Toxicology and Ecology, GB/TB - Z470, 67056 Ludwigshafen, Germany; 2 Scientific Consultancy – Animal Welfare, Hallstattfeld 16, 85579 Neubiberg, Germany; 3 BASF Schweiz AG, GUP/PS - K141, 4057 Basel, Switzerland; 4 BASF SE, Material Physics and Analytics, GMC/R - B7, 67056 Ludwigshafen, Germany

**Keywords:** Nanomaterials, Bovine Corneal Opacity and Permeability assay (BCOP), EpiOcular™ Eye Irritation Test (EIT), Severe eye damage, Eye irritation, In vitro

## Abstract

**Background:**

Assessment of eye irritation hazard has long been a core requirement in any chemical legislation. Nevertheless, publications focussing on the eye damaging potential of nanomaterials are scarce. Traditionally, eye irritation testing was performed using rabbits. The OECD Test Guideline 437 *Bovine Corneal Opacity and Permeability (BCOP) test method* allows determining severely irritating substances without animals, and the recently adopted OECD Test Guideline 492 *Reconstructed human cornea-like epithelium test method* allows identifying chemicals that neither induce eye irritation nor serious eye damage. For substances applicable to these tests, huge progress has been made in replacing animal testing.

**Methods:**

The in vitro eye irritation potential of 20 nanosized and 3 non-nanosized materials was investigated in a 2-tier EpiOcular™ Eye Irritation Test (EpiOcular™-EIT) and BCOP testing strategy including histopathology of the bovine corneas. Furthermore, applicability of the testing strategy for nanomaterials was assessed. Test materials encompassed OECD representative nanomaterials (metals (Ag), metal oxides (ZnO, TiO_2_, CeO_2_), amorphous SiO_2_ and MWCNTs), three organic pigments, quartz, and talc.

**Results:**

None of the dry-powder nanomaterials elicited eye irritation in either the EpiOcular™-EIT or the BCOP assay. Likewise, an amorphous SiO_2_ nanomaterial that was supplied as suspension was tested negative in both assays. By contrast, in the EpiOcular™-EIT, the silver nanomaterial that was supplied as dispersion was tested positive, whereas its surfactant-containing dispersant was borderline to negative. In the BCOP assay, the silver nanomaterial elicited highly variable results and dark-brown patches remained on the corneal surface, whereas the results for its dispersant alone were borderline to positive, which was assessed as inconclusive due to high inter-assay variability.

**Conclusion:**

The present study points to the low eye irritation potential of a spectrum of nanomaterials, which is consistent with available in vivo data for the same test materials or for nanosized or bulk materials of the same composition.

**Electronic supplementary material:**

The online version of this article (doi:10.1186/s12989-016-0128-6) contains supplementary material, which is available to authorized users.

## Background

Throughout the world, assessment of potential eye irritation hazard has long been a core requirement in chemical legislation. For instance, information on this endpoint is mandatory for all (nanosized or conventional) substances in accordance with the European Regulation on the Registration, Evaluation, Authorisation, and Restriction of Chemicals (REACH [1]). To fulfil global legislative requirements, testing protocols adopted by the Organisation for Economic Co-operation and Development (OECD) are needed. As a result, the majority of existing assessments are based on animal tests (i.e. OECD Test Guideline (TG) 405 [2]).

With the adoption of the TG for the *Bovine Corneal Opacity and Permeability (BCOP) test method for identifying ocular corrosives and severe irritants* (OECD TG 437) in 2009, it became possible to register severely irritating substances (i.e. ‘Category 1’ substances; *cf.* Information box on hazard categorization) without the need for animal testing. In 2013, the TG for the BCOP assay was revised to further allow identifying substances not requiring classification for eye irritation or serious eye damage, i.e. ‘Non-category’ substances if the irritation scores are below a certain threshold limit [3]. Finally, with the recent adoption of the TG for the *Reconstructed human cornea-like epithelium test method for identifying chemicals not requiring classification and labelling for eye irritation or serious eye damage* (OECD TG 492 [4]) on 28 July 2015, during the preparation of the present article, huge progress has been made in replacing animal testing for the endpoint ‘eye irritation’.

**Table:** 

**Information box - Hazard categorization of substances causing local ocular toxicity** The **European Regulation on the Classification, Labelling and Packaging (CLP) of Substances and Mixtures** [5] distinguishes two categories: - Category 1 for substances causing serious or irreversible eye damage (‘corrosion’) that persists within 21 days post-exposure; - Category 2 for those inducing reversible eye damage (‘irritation’). Specifically, Category 2 is assigned if a substance produces - at least in 2 of 3 tested animals - a positive response of corneal opacity ≥1 and/or iritis ≥1 and/or conjunctival redness ≥2 and/or conjunctival edema (chemosis) ≥2 (calculated as the mean scores following grading at 24, 48 and 72 h after treatment) and all effects are fully reversible within 21 days [5]. The **United Nations Globally Harmonised System** (UN GHS [6]) further foresees differentiating between - Category 2A (irritating to the eye) - Category 2B (mildly irritating to the eye), if effects are fully reversible within 7 days of substance application.	

Notwithstanding the human health relevance of this endpoint, publications focussing on the eye damaging potential of nanomaterials (NMs) are scarce. The majority of publications addressing their toxicological effects, either in vivo or in vitro, report investigations on respiratory tract effects, since for most NMs, inhalation is the predominant route of exposure provided that they are released either during production or use [7, 8]. Most of the available studies on eye irritation evaluated the local toxicity of NMs developed for intentional application to the eye, e.g. as ocular drug delivery devices, a topic reviewed by Prow [9]. Frequently, the safety of such devices is assessed in in vivo studies using rabbits [10–15]. As regards in vitro eye irritation testing of nanosized ocular drug carriers [16, 17], recently, Bhasker et al*.* [18] evaluated the effects of nanoformulated bovine lactoferrin or SurR9-C84A proteins on normal or insulted *ex vivo* bovine cornea models observing eye-irritating properties on neither model. Generally, the eye irritation potential of ocular drug carriers appears to increase with increasing lipophilicity as was assessed using a set of different non-nanosized lipoamino acids in saline solution [19].

Even fewer scientific publications are available addressing the eye irritation potential of NMs that are not intended for topical ocular application: In an in vivo rabbit eye irritation study performed in accordance with the OECD TG 405, 21 % anatase / 79 % rutile TiO_2_ (140 nm in water; Dynamic Light Scattering (DLS)) produced reversible conjunctival redness [20]. Instilling 5,000 ppm self-prepared precipitated colloidal Ag NMs (10-20 nm) in aqueous suspension into the eyes of guinea pigs produced eye irritation that was fully reversible within 24 h [21]. In humans, silver particles from silver-containing antibacterial agents were found to deposit in the cornea [22]. Kishore et al*.* [23] reported two differently sized multi-walled carbon nanotubes (MWCNTs) to elicit reversible conjunctival redness and discharge in the rabbit eye (MWCNT 1: 5–8 μm length; 3–8 nm inside diameter; 140 ± 30 nm outside diameter; MWCNT 2: 1–10 μm length; 2–6 nm inside diameter; 10–15 nm outside diameter; both MWCNTs forming compact aggregates). While the limited data available in the published literature suggest that NM eye irritancy potential might generally be low, there are currently no studies allowing the comparison of ocular effects induced by different types of NMs.

Therefore, in the present study the in vitro eye irritation potential of a broad spectrum of altogether 20 nanosized and three non-nanosized materials was investigated. Sixteen NMs were OECD representative NMs that are metals (Ag), metal oxides (ZnO, TiO_2_, CeO_2_), precipitated and pyrogenic amorphous SiO_2_ and MWCNTs. These NMs have been coded in the list of the OECD Sponsorship Program for the Testing of Manufactured Nanomaterials (http://www.oecd.org/science/nanosafety/; accessed 7 April 2016). Since Ag NM-300 K was provided as dispersion, also its dispersant alone, i.e. Ag NM-300 K DIS, was assessed (NM-x numbers refer to the respective codes of the OECD representative NMs). Since all further OECD representative NMs were provided as powder, Levasil® 200, a precipitated amorphous SiO_2_ that is provided as 40 % suspension (in the following: aSiO_2_-susp), was included as further test material to evaluate whether the ‘as supplied’ preparation of a test material affects eye irritation potential or potency. Likewise, non-nanosized quartz dust DQ12 was taken up into the study, since crystalline SiO_2_ is known to generally have a higher toxic potential than amorphous SiO_2_. Additionally, three organic pigments that fall under the EU recommendation on the definition of nanomaterials [24] representing three different chemical classes, i.e. Pigment Red 57:1, Pigment Yellow 95, and Pigment Black 32, were included. These pigments were selected based upon availability of physico-chemical characterisation data and Good Laboratory Practice-compliant animal data. To date, no organic pigment has been classified as an eye irritant. Otherwise it would have been included in the present study. Finally, non-nanosized talc, the historical negative control for in vivo eye irritation testing, was added to the spectrum of test materials.

All 23 test materials were submitted to a 2-tier non-animal testing strategy composed of the EpiOcular™ Eye Irritation Test (EpiOcular™-EIT; OECD TG 492) and the Bovine Corneal Opacity and Permeability (BCOP; OECD TG 437) assay including histopathological evaluation of the cornea [25, 26]. Thereby, the present study further aimed at investigating the usefulness of this testing strategy for the hazard assessment and categorization of NMs. Of note, while histopathological evaluation of the cornea has not yet been incorporated in OECD TG 437, the OECD has published a guidance document to promote such assessments [27]. The outcome of the in vitro 2-tier testing was compared to available in vivo data from eye irritation studies performed in accordance with OECD TG 405 (or comparable methods). For the three organic pigments, in-house data from earlier in vivo eye irritation studies were available. For the other test materials, in vivo eye irritation data and classifications from corresponding REACH dossiers were evaluated. No new in vivo studies were performed for the purpose of the present study.

Whereas data from in vitro eye irritation tests may be used to fulfil the REACH standard information requirements for substances with low annual production volumes, before the adoption of OECD TG 492 in July 2015, the in vivo rabbit eye irritation test was generally prescribed for all substances with annual volumes exceeding 10 tonnes [1] unless the existing in vitro data were suitable for hazard classification and labelling. The animal welfare concerns and scientific limitations of this so-called ‘Draize test’ have been widely recognized for many years [28]. However, in spite of numerous research efforts [29], on the global scale its full replacement in accordance with the 3Rs principle [30, 31] is outstanding.

The BCOP assay (OECD TG 437), as partial replacement method, allows the identification of ‘Non-category’ substances and ‘Category 1’ substances inducing serious or irreversible eye damage. To date, there is no non-animal test method allowing direct ‘Category 2’ classification for eye irritation having achieved regulatory acceptance. However, human cornea-like three-dimensional (3D) reconstructed tissue models are promising in vitro models to distinguish ‘Non-category’ substances from ‘Category 1 or 2’ substances eliciting corrosive or irritating effects [25, 32, 33]. An example for such an assay is the EpiOcular™-EIT. It uses the cornea-like non-keratinized tissue construct EpiOcular™ (MatTek Corp., USA) that is composed of normal human epidermal keratinocytes obtained from individual donors. The model is cultured in proprietary serum-free culture medium which induces corneal differentiation and formation of the organotypic cornea-like model. This three dimensional tissue consists of highly organized cell layers and exhibits barrier properties similar to the normal in vivo corneal epithelium [32]. Tissue destruction is determined by formazan reduction after incubation with the tetrazolium salt 3-[4,5-dimethylthiazol-2-yl]-2,5-diphenyltetrazolium bromide (MTT), which reflects impaired mitochondrial dehydrogenase activity.

The concept of combining methods to identify ocular non-irritants and substances causing serious eye damage to cover the full spectrum of eye irritation has previously been described by Scott et al*.* [34], and the specific combination of the EpiOcular™-EIT and the BCOP assay has been submitted to an in-house validation study in the authors’ laboratory [25]. Accordingly, with the adoption of OECD TG 492, substances applicable to the two in vitro test methods (i.e. the EpiOcular™-EIT and the BCOP assay) should no longer require animal testing, since ‘Category 2’ substances may be assessed indirectly: In the 2-tier ‘bottom up’ testing strategy we suggest, the EpiOcular™-EIT is performed in Tier 1 to distinguish ‘Category 1 or 2’ from ‘Non-category’ substances. In Tier 2, the BCOP assay is conducted to identify ‘Category 1’ substances from within the set of ‘Category 1 or 2’ substances identified in Tier 1. All substances that are not identified as ‘Category 1’ in the BCOP assay in Tier 2 are classified as ‘Category 2’. By contrast, in a ‘top down’ approach, a substance considered a severe ocular irritant would undergo the BCOP assay first, and if the result would indicate that the substance is not ‘Category 1’ after all, it would undergo the EpiOcular™-EIT [25, 34]. However, also in the light of the newly adopted OECD TG 492 [4], the European Chemicals Agency (ECHA) holds a different view on the comprehensiveness of in vitro testing. In the October 2015 version of the ECHA guidance on endpoint-specific information requirements, a footnote in the section on serious eye damage/eye irritation states: *Please note that the information requirements in REACH Annexes VII and VIII* [i.e. the standard information requirements for subst*ances imported or manufactured at production volumes of 1 or 10 tons or more, respectively] in relation to skin corrosion/irritation and serious eye damage/eye irritation are currently under revision. This revision is expected to strengthen the role of in vitro methods and to remove the standard information requirement for an in vivo study at the Annex VIII level. As a consequence, once the new REACH Annexes come into force, an in vivo study would only be required where a substance falls outside of the applicability domain of the available in vitro methods or the results obtained from such methods would not allow a conclusive decision on (non-) classification and risk assessment* [35].

To the best of the authors’ knowledge, to date, there are no publications reporting the in vitro assessment of NMs intended for non-medicinal applications in the BCOP assay (OECD TG 437) or the EpiOcular™-EIT (OECD TG 492). In accordance with the 3R principle to refine, reduce and eventually replace animal tests [30] in the present study, the 2-tier EpiOcular™- BCOP eye irritation testing strategy was applied to assess the eye irritation potential of the nanosized and non-nanosized test materials. Therefore, the present study, submitting a broad panel of extensively characterized NMs to in vitro eye irritation and corrosion testing, not only aimed at comparing the eye irritancy potential of these materials. In the light of the view of the OECD Working Party on Manufactured Nanomaterials (WPMN) that modifications to address the NM specificities of existing OECD TGs [36] may be required, this study further served to provide a first understanding on the applicability of these two OECD TGs for the testing of NMs.

## Methods

### Test materials and particle characterisation

The 16 OECD representative NMs were supplied by the European Commission’s Joint Research Centre (Italy). They comprised six different TiO_2_ (anatase NM-100, NM-101, NM-102; rutile NM-103, NM-104; and rutile-anatase NM-105), uncoated (NM-110) and coated (NM-111) ZnO, amorphous SiO_2_ produced by precipitation (NM-200) and by thermal process (NM-203), uncoated CeO_2_ produced by precipitation (NM-211, NM-212), monodispersed Ag produced by precipitation (NM-300 K), and three MWCNTs of different lengths and diameters (NM-400, NM-401, NM-402). All OECD representative NMs were provided as powder, except for Ag NM-300 K, which was provided in dispersion to prevent its spontaneous oxidation in air. The Ag NM-300 K dispersant is an aqueous solution containing the capping agents polyoxyethylene glycerol trioleate and polyoxy ethylene-(20) sorbitan mono-laurate (Tween 20). This dispersant was included as a further, 17^th^ (non-nanosized) test material (NM-300 K DIS) to verify whether possible effects induced by Ag NM-300 K were caused by the silver or by its dispersant.

aSiO_2_-susp was supplied by AkzoNobel AB (Sweden) and quartz dust DQ 12 by Doerentrup Quarz GmbH (Germany). Talc was purchased from a local retailer, and the three organic pigment samples of commercial grade were provided by BASF SE.

Table 1 presents details of the intrinsic material properties of the test materials as well as the eye irritation classifications from corresponding REACH dossiers (www.echa.europa.eu/information-on-chemicals; accessed 7 April 2016). For Pigment Red 57:1, a total number of 13 in vivo studies were identified during the preparation of the REACH registration dossier. Pigment Yellow 95 and Pigment Black 32 had each been tested in house in a Good Laboratory Practice-compliant in vivo study (OECD TG 405).

**Table 1:** Intrinsic material properties of the test substances (for the OECD representative NMs, as provided by the supplier ^a^)

OECD no./substance name	Test material	Physical state/form of production	Purity (%), impurities	Particle size (nm) and shape	SSA (BET; m^2^/g)	Classification ‘serious eye damage/eye irritation’ ^b^ (CAS No./EC List No.)
NM-100	TiO_2_ pigmentary	Anatase, uncoated	>98.5	TEM: 42–90	10	Conclusive, but not sufficient for classification (CAS No. 13463-67-7)
NM-101	TiO_2_	Anatase, uncoated	91.7	8, XRD: 6	320	
NM-102	TiO_2_	Anatase, uncoated	96.0	22, XRD: 20	90	
NM-103	TiO_2_	Rutile, ultrafine, hydrophobic	89.0; Al_2_O_3_: 6.2 %	20, XRD: 20	60	
NM-104	TiO_2_	Rutile, ultrafine, hydrophilic	89.8; Al_2_O_3_: 6.2 %	20, XRD: 20	60	
NM-105	TiO_2_	Rutile-anatase, uncoated	> 99	21, XRD: 22	61	
NM-110	ZnO	Uncoated, white powder	> 99	XRD: 41.5, spherical	13	ZnO nano: Conclusive, but not sufficient for classification (CAS No. 1314-13-2)
NM-111	ZnO	Coated with 1–4 % triethoxycaprylyl silane, white powder	96–99	XRD: 33.8, spherical	16	
NM-200	Amorphous SiO_2_	Produced by precipitation, white powder	96.5	TEM: 20, spherical	230	Amorphous fumed silica and precipitated silica gel: Conclusive, but not sufficient for classification (CAS No. 7631-86-9)
NM-203	Amorphous SiO_2_	Produced by thermal process, white powder	-	TEM: 20, spherical, irregular	226	
aSiO_2_-susp	Amorphous SiO_2_	Synthesis by means of a growth process from an aqueous solution with dissociated molecular silicic acid, opalescent suspension	>99 %	TEM: 15	200	
Quartz dust DQ12	Crystalline SiO_2_	Powder	87 %; rest: amorphous SiO_2_, 0.2 % Al_2_O_3_; 0.03 % Fe_2_O_3_	89, hexagonal	5.9	
NM-211	CeO_2_	Uncoated, produced by precipitation, yellowish powder	> 95	XRD: 10.3, cubic	66	Bulk cerium dioxide: Conclusive, but not sufficient for classification (CAS No. 1306-38-3)
NM-212	CeO_2_	Uncoated, produced by precipitation, yellowish powder	> 99.5	XRD: 33, cubic	28	
NM-300 K	Silver < 20 nm	Produced by chemical precipitation of AgNO_3_, monodispersed with capping agents (cf. NM-300 K DIS); silver content: 10.16 % w/w, very viscous, concentrate orange-brown, yellow in dilution	10	TEM: 15, colloidal, spherical	Not available	Silver (≥ 99.9 % Ag in nano form; median particle size <100 nm): Conclusive, but not sufficient for classification (CAS No. 7440-22-4)
NM-300 K DIS	Ag dispersant	Aqueous solution containing the capping agents: 4 % each of polyoxyethylene glycerol trioleate and polyoxyethylene (20) sorbitan mono-laurate (Tween 20), very viscous				Tween 20 (CAS No. 9005-64-5) non-irritant (OECD TG 437)
NM-400	MWCNT	Specialty graphite, produced by CCVD, at 20 °C and 1013 hPa: solid powder, black odourless, insoluble in water or organic solvent, surface charge: NA, conductive	> 90; impurities: < 10 % metal oxide	TEM: Ø 9.5, 1.5 μm length, short, thin, tangled, concentric tubes	280	Conclusive, but not sufficient for classification (EC List No. 936-414-1)
NM-401	MWCNT	Specialty graphite, produced by CCVD, at 20 °C and 1013 hPa: solid powder, black odourless, insoluble in water or organic solvent, surface charge: NA, conductive	> 95, impurities: < 5 % metal oxides	TEM: Ø 10 - 30, 5 - 15 μm length, short, thin, tangled, concentric tubes	300	
NM-402	MWCNT	Specialty graphite, produced by CCVD, at 20 °C and 1013 hPa: solid powder, black odourless, insoluble in water or organic solvent, surface charge: NA, conductive	> 90, impurities: < 10 % metal oxides	TEM: Ø 5 - 15, 0.1 – 10 μm length, short, thin, tangled concentric tubes	250–300	
Talc		Powder	>97 %	non-nanosized	non-nanosized	Exempt from REACH registration (CAS No. 14807-96-6)
Pigment Red 57:1		Red powder, insoluble in water and octanol	>90 %	TEM: polydisperse from 20 nm to 200 nm, mostly irregular shapes of low aspect ratio, some rods	59	Not irritating; 13 eye irritation studies available, mostly comparable to OECD TG 405, some GLP-compliant (CAS No. 5281-04-9)
Pigment Yellow 95		Yellow Powder, insoluble in water and octanol	>99 %	TEM: mostly rods with aspect ratios around 5, diameters from 30 nm to 150 nm	57	Not irritating (OECD TG 405, GLP-compliant)
						Average score (24–72 h) for irritation: 0.0 for iris, 0.1 for corneal opacity and conjunctival redness, and 0.3 for chemosis. (CAS No. 5280-80-8)
Pigment Black 32		Black Powder, insoluble in water and octanol	>99 %	TEM: mostly rods with aspect ratios around 5, diameters from 40 nm to 200 nm	39	Not irritating (OECD TG 405, GLP-compliant)
						Average score (24–72 h) for irritation: 0.0 for corneal opacity, iris and chemosis and 0.3 for conjunctival redness.(EC List No. 475-310-6)

Abbreviations: *BET* (Method of) Brunauer-Emmett-Teller, *CCVD* Catalytic chemical vapour deposition, *GLP* Good laboratory practice, *MPS* mean particle size, *MWCNT* Multi-walled carbon nanotube, *PPS* primary particle size, *SSA* Specific surface area, *TEM* Transmission electron microscopy, *XPS* X-ray photoelectron spectroscopy, *XRD* X-ray diffraction

^a^ For the OECD representative NMs, the European Commission’s Joint Research Centre, Ispra, Italy (Table parts referring to the 16 OECD representative NMs adapted from Sauer et al*.* [37]); for quartz dust DQ12, Dörentrup Quarz GmbH & Co. KG, Germany, www.doerentrup.de (and further from Wohlleben et al*.* [70])

^b^ Eye irritation classifications were retrieved from the corresponding REACH dossiers (www.echa.europa.eu/information-on-chemicals; accessed 7 April 2016). The formal phrase *conclusive but not sufficient for classification* implies that the substance was not classified as hazardous for this endpoint

The particle sizes of the OECD representative NMs suspended in water (as they were prepared for application in the BCOP assay; albeit excluding ZnO NM-111 and MWCNT NM-401 and NM-402) as well as details on their water solubility are presented in Table 2. Particle sizes in water were determined by two complementary techniques, i.e. Analytical Ultracentrifugation (AUC) and Laser Diffraction (LD). AUC relies on the time- and space-resolved observation of sedimentation processes and allows quantifying the fractions of the total dose that are dispersed to diameters <100 nm and <1000 nm, respectively. LD relies on the optical observation of the diffraction and scattering of light, and provides the median diameter (LD D_50_) of the agglomerate fraction. LD, however, is not reliable to detect dispersed nanoparticles. Therefore, in the present study, prevalence was given to AUC to determine which NMs were dispersed with significant fractions below 100 nm. If results from the AUC indicate that even the size interval up to 1000 nm only contains a smaller part (i.e. <50 %) of the total dose of a given NM, this NM is strongly agglomerated with certainty. In these cases, the LD D_50_ values are most likely reliable representations of the average agglomerate particle size. If, however, results from the AUC indicate that the size interval up to 1000 nm contributes considerably to the total dose, the LD D_50_ values have to be evaluated with caution, since they will not represent both small and large particles.

**Table 2:** Solid test substances ^a^: Particle size in a 200 mg/mL suspension in water (applied in the BCOP assay), determined by analytical ultracentrifugation (AUC) and laser diffraction (LD)

OECD number	Test material	Particles <1000 nm [mg/mL; AUC]	Particles <100 nm [mg/mL; AUC]	D_50_ [μm; LD]	Water solubility [AUC combined with ICP-MS / ICP-AES]
NM-100	TiO_2_ pigmentary	249 ^b^	8.6	0.374	
NM-101	TiO_2_	101	3.3	0.959	
NM-102	TiO_2_	13.7	0.0	1.23	
NM-103	TiO_2_	69.2	0.0	1.56	
NM-104	TiO_2_	131	0.8	1.10	
NM-105	TiO_2_	73.2	0.2	0.938	Wohlleben et al*.* [70]: Ti < 0.1 ppm
NM-110	ZnO	27.4	0.0	3.00	Wohlleben et al*.* [70]: Only soluble in acidic environments
NM-200	Amorphous SiO_2_	11.9	0.0	11.6	OECD [71]: 2.4 ± 0.03 mmol/l (corresponding to 67 mg/L)
NM-203	Amorphous SiO_2_	132	92.6	21.2	Wohlleben et al*.* [70]: Si: 56 ppm
NM-211	CeO_2_	62.6	5.8	2.14	Wohlleben et al*.* [70]: CeO_2_: < 0.1 ppm
NM-212	CeO_2_	132	0.3	0.776	Keller et al*.* [72]: 0.002 wt.%
NM-400	MWCNT	14.8	0.0	383	Insoluble, supernatant fully transparent

^a^ For technical reasons (see text for details), ZnO NM-111 and MWCNT NM-401 and NM-402 were exempt from the characterization. Additionally, the substances that were applied undiluted in the BCOP assay were exempt from the characterization

^b^ The recorded value exceeds the total dose. Possibly, the refractive index increment for TiO_2_ (dn/dc = 0.376 cm^3^/g) does not apply for TiO_2_ NM-100, e.g. due to its exclusively anatase crystal structure

For further details on the material properties of the 16 OECD representative NMs, *cf.* Sauer et al*.* [37, 38].

### Test material preparation

#### EpiOcular™-EIT

In the EpiOcular™-EIT, the test materials were applied undiluted at amounts enabling to cover the entire tissue surfaces. As a rule, this corresponded to 50 μL bulk volume of the respective dry-powder test material or 50 μL of the liquid test materials Ag NM-300 K, Ag NM-300 K DIS and aSiO_2_-susp. Only the MWCNTs had to be applied at higher volumes to enable covering the entire EpiOcular™ tissue surfaces (*cf.* Tables 3, 4 and 5 for the respective NM masses applied).

**Table 3:** Mean tissue viability of 16 OECD representative nanomaterials (and the Ag NM dispersant) determined in the EpiOcular™ eye irritation test (protocol variant 1 ^a^)

Test material	TiO_2_ NM-100	TiO_2_ NM-101	TiO_2_ NM-102	TiO_2_ NM-103	TiO_2_ NM-104	TiO_2_ NM-105	ZnO NM-110	ZnO NM-111	SiO_2_ NM-200	SiO_2_ NM-203	CeO_2_ NM-211	CeO_2_ NM-212	Ag NM-300 K	Ag dispersant NM-300 K DIS	MWCNT NM-400	MWCNT NM-401	MWCNT NM-402
Volume or mass applied	28 mg ^b^	22 mg ^b^	23 mg ^b^	17 mg ^b^	12 mg ^b^	6 mg ^b^	12 mg ^b^	25 mg ^b^	6 mg ^b^	2.5 mg ^b^	61 mg ^b^	28 mg ^b^	50 μL	50 μL	46 mg ^c^	32 mg ^c^	8 mg ^c^
Viability of tissue #1 relative to NC	99	107	105	79	70	97	96	116	107	88	92	81	42	62	88	109	98
Viability of tissue #2 relative to NC	83	107	106	79	86	106	100	113	105	91	70	81	48	71	95	105	100
Mean relative tissue viability (and ITV%)	91 (16)	107 (1)	106 (1)	79 (0)	78 (16)	102 (9)	98 (5)	114 (3)	106 (2)	90 (3)	81 (22 ^d^)	81 (0)	45 (6)	66 (9)	92 (7)	107 (4)	99 (2)

The mean tissue viability of 2 tissues / test group is expressed relative to the corresponding negative control (NC) value further indicating (in brackets) the relative inter-tissue variability (ITV%)

^a^ Protocol variant 1: 30 min and 90 min exposure duration for liquids and solids, respectively; 12 min post-exposure immersion for both liquids and solids

^b^ Corresponding to 50 μL bulk volume

^c^ Corresponding to 2×50 μL bulk volume

^d^ Since the inter-tissue variance of the two tissues was >20 %, the corresponding acceptance criterion was not met. However, since all other acceptance criteria were met and due to the unambiguous result recorded for the test substance, the test was considered valid despite this deviation and therefore was not repeated

**Table 4:** Mean tissue viability of 15 OECD representative nanomaterials determined in the EpiOcular^TM^ eye irritation test (protocol variant 2, performed with solid test substances only and excluding MWCNT NM-402 ^a^)

Test material	TiO_2_ NM-100	TiO_2_ NM-101	TiO_2_ NM-102	TiO_2_ NM-103	TiO_2_ NM-103	TiO_2_ NM-103	TiO_2_ NM-104	TiO_2_ NM-105	ZnO NM-110	ZnO NM-110	ZnO NM-111	ZnO NM-111	SiO_2_ NM-200	SiO_2_ NM-200	SiO_2_ NM-203	SiO_2_ NM-203	CeO_2_ NM-211	CeO_2_ NM-211	CeO_2_ NM-212	MWCNT NM-400	MWCNT NM-401
No. of test run				1^st^ test run ^b^	2^nd^ test run ^b^	3^rd^ test run ^b^			1^st^ test run ^b^	2^nd^ test run	1^st^ test run^b^	2^nd^ test run	1^st^ test run^c^	2^nd^ test run	1^st^ test run ^c^	2^nd^ test run	1^st^ test run ^c^	2^nd^ test run			
Volume or mass applied	28 mg ^d^	22 mg ^d^	23 mg ^d^	17 mg ^d^	17 mg ^d^	17 mg ^d^	12 mg ^d^	6 mg ^d^	12 mg ^d^	12 mg ^d^	25 mg ^d^	25 mg ^d^	6 mg ^d^	6 mg ^d^	2.5 mg ^d^	2.5 mg ^d^	61 mg ^d^	61 mg ^d^	28 mg ^d^	46 mg ^e^	32 mg ^e^
Viability of tissue #1 relative to NC	108	92	96	30	41	102	89	98	74	63	9	87	127	125	120	81	128	113	113	115	124
Viability of tissue #2 relative to NC	107	97	115	61	80	29	107	121	115	73	131	94	134	95	137	111	131	105	97	106	117
Mean relative tissue viability (and ITV%)	108 (1)	95 (5)	106 (20 ^f^)	45 (31)	60 (40)	66 (73)	98 (17)	109 (23 ^f^)	94 (40)	68 (10)	114 (34)	91 (7)	131 (7)	110 (30)	128 (17)	96 (30)	129 (3)	109 (8)	105 (16)	111 (9)	120 (7)

The mean tissue viability of 2 tissues / test group is expressed relative to the corresponding negative control (NC) value further indicating (in brackets) the relative inter-tissue variability (ITV%)

^a^ Protocol variant 2 (performed only with solid test substances): 6 h exposure duration for solids; 25 min post-exposure immersion for solids

^b^ Repeat of test run due to high variability >20 % (in the case of TiO_2_ NM-103: Cessation of testing after 3^rd^ inconclusive test run)

^c^ Repeat of test run due to high optical density value (>130 % relative to the NC) of one of the measurements

^d^ Corresponding to 50 μL bulk volume

^e^ Corresponding to 2x50 μL bulk volume

^f^ Even though the ITV% values laid close to the threshold value of 20 %, these test runs were not repeated, since the individual measurements clearly indicated lack of toxicity

**Table 5:** Mean tissue viability of aSiO_2_-susp, quartz dust DQ12, talc, and three organic pigments determined in the EpiOcular™ eye irritation test (protocol variant 2 for solid test substances ^a^)

Test substance	aSiO_2_-susp	aSiO_2_-susp	Quartz dust DQ12	Pigment Red 57:1	Pigment Yellow 95	Pigment Black 32	Talc
No. of test run	1^st^ test run	2^nd^ test run ^b^					
Volume or mass applied	50 μL	50 μL	15 mg ^c^	12 mg ^c^	9 mg ^c^	8 mg ^c^	16 mg ^c^
Viability of tissue #1 relative to NC	67	89	73	98	87	97	93
Viability of tissue #2 relative to NC	90	107	82	98	95	79.6	103
Mean relative tissue viability (and ITV%)	78 (23)	98 (18)	77 (10)	98 (0)	91 (8)	88 (18)	98 (10)

The mean tissue viability of 2 tissues / test group is expressed relative to the corresponding negative control (NC) value further indicating (in brackets) the relative inter-tissue variability (ITV%)

^a^ Protocol variant 2 for solid test substances: 6 h exposure duration for solids; 25 min post-exposure immersion for solids

^b^ Repeat of test run due to high variability >20 %

^c^ Corresponding to 50 μL bulk volume

#### BCOP assay

The OECD representative NMs that had been delivered as powders (i.e. all except Ag NM-300 K and its dispersant) were suspended in highly de-ionized water to achieve final concentrations of 20 % (w/v) suspension and then stirred with a magnetic stirrer. Additionally, TiO_2_ NM-103, TiO_2_ NM-104, ZnO NM-110, and SiO_2_ NM-200 were stirred with a high-speed homogenizer (Ultra-Turrax, Jahnke & Kunkel (IKA-Werke), Germany). All test material suspensions were applied immediately after preparation, and also during test material application, the preparations were stirred with a magnetic stirrer to ensure continued homogeneity. Suspensions of ZnO NM-111 and MWCNT NM-401 could not be prepared homogeneously. Hence, these test materials were applied undiluted, just as Ag NM-300 K, Ag NM-300 K DIS, aSiO_2_-susp, quartz dust DQ12, the three organic pigments, and talc were applied undiluted.

### In vitro testing

#### EpiOcular™-EIT

The EpiOcular™-EIT was performed in two variants. The 16 OECD representative NMs and Ag NM-300 K DIS were submitted to protocol variant 1, i.e. as described by the supplier MatTek [39] and Harbell et al*.* [40]. aSiO_2_-susp, quartz dust DQ12, the three organic pigments, and talc were assessed in accordance with variant 2, i.e. as described in the OECD TG 492. To ensure data comparability, the dry-powder OECD representative NMs (excluding MWCNT NM-402) were additionally submitted to variant 2. The two test protocol variants differ in respect to the exposure and post-exposure immersion periods laid down for solid test materials. In the following, both EpiOcular™-EIT protocol variants are presented jointly specifically indicating the different exposure and post-exposure durations.

##### Reagents, test systems, and technical equipment, positive and negative controls

The following reagents, test systems and technical equipment were used: Spectrophotometer: Sunrise™ Absorbance Reader (Tecan Group Ltd., Switzerland; measurement using a filter wavelength of 570 nm without reference filter). MatTek Corp. (USA) and MatTek In Vitro Life Science Laboratories (Slovakia) provided the following reagents and test systems: EpiOcular™ OCL-200 kit (containing 24 OCL-200 reconstructed cornea tissues, 0.6 cm^2^ surface area, cultured in Millicells® with 1-cm diameter); Dulbecco‘s modified eagle’s medium (DMEM; also purchased from Sigma Aldrich, Germany); Dulbecco’s phosphate buffered saline (PBS) without Ca^2+^ or Mg^2+^ (also purchased from Biochrom, Germany); MTT (also purchased from Sigma Aldrich, Germany), and 1.0 mg/mL isopropanol.

Highly de-ionized water was used as negative control (NC) and methyl acetate (purity >98 %, Chemical Abstracts Service (CAS) No. 79–20–9, Merck KGaA, Germany) as positive control (PC).

##### Pre-tests to determine direct MTT reduction

For all test materials, *pre-tests* as described in the OECD TG 492 were performed that precluded the test materials’ ability to directly reduce MTT.

##### Main tests

In the *main tests*, two tissues were treated with either the test materials, the NC or the PC. On the day of arrival in the laboratory, the EpiOcular™ tissues were transferred to sterile 6-well plates with 1 mL DMEM and pre-conditioned at standard culture conditions (37 °C, 5 % CO_2_, 90–95 % humidity) in the incubator for 16–24 h. After pre-incubation, the tissues were pre-treated with 20 μL PBS and further incubated at standard culture conditions for 30 min. Using a sharp spoon or pipette, the dry-powder or liquid test items, respectively, were applied to cover the entire tissue surface (*cf.* 2.2.1). Control tissues were concurrently exposed to 50 μL highly de-ionized water (NC) or methyl acetate (PC). After test material application, the tissues were placed into the incubator for the following *exposure periods*: 90 min (solids; variant 1), 6 h (solids; variant 2), or 30 min (liquids).

To remove the test materials, the tissues were washed with sterile PBS and immediately immersed into 12-well plates, pre-filled with 5 mL pre-warmed medium per well to remove test material residuals. After 12 min (solids, variant 1; and liquids) or 25 min (solids; variant 2), each tissue was dried on absorbent paper and transferred to fresh 6-well plates filled with 1 mL pre-warmed medium per well (*post-exposure immersion*). Subsequently, the tissues were incubated at standard culture conditions (*post-exposure incubation*) for 18 h (solids; variants 1 and 2) or 2 h (liquids). During the post-exposure immersion and incubation periods, weak cytotoxic effects might reverse, and more pronounced effects might increase.

Upon completion of the respective post-exposure periods, the assay medium was replaced by 0.3 mL MTT solution. After incubating the tissues for 3 h, the tissues were washed with PBS to terminate the MTT incubation. The produced formazan was extracted by incubating the tissues in isopropanol at room temperature overnight or on a plate shaker for at least 2 h. The optical density of the formazan extracts was determined spectrophotometrically at a wavelength of 570 nm (OD_570_). For each microtitre plate, blank values were established from 4 wells filled with isopropanol.

##### Calculation of mean relative tissue viability

Tissue OD_570_ values were calculated by subtracting the mean blank value of the respective microtitre plate from the measured tissue OD_570_ value, and mean OD_570_ values were calculated for the two tissues of each treatment group. The quotient of the mean OD_570_ values of the test material-treated tissues and those of the NC (i.e. the *mean relative tissue viability*) was determined to evaluate whether or not a test material is an irritant:

- Mean relative tissue viabilities ≤60 % indicated ‘irritancy to the eye’;
- Mean relative tissue viabilities >60 % indicated ‘no irritancy to the eye’.

##### Acceptance criteria

In case one of the following acceptance criteria (AC) as described in the OECD TG 492 was not met, repetition of the EpiOcular™-EIT was considered.

- AC for the NC: The OD_570_ of the NC reflects the laboratory-specific tissue viability under the specific conditions of the assay. It was considered acceptable if the mean OD_570_ of the NC was ≥0.8 and ≤2.5 and the historical in-house mean at the respective time of testing was met (variant 1: OD_570_ of NC for liquids / solids: 1.490 ± 0.106 / 1.361 ± 0.138; variant 2: OD_570_ of NC for solids: 1.650 ± 0.159).
- AC for the PC*:* In-house, the PC methyl acetate usually elicits relative tissue viabilities of approx. 25 % (historical in-house means at the time of testing in accordance with variant 1: OD_570_ of PC for liquids/solids: 0.388 ± 0.098/0.318 ± 0.119; variant 2: OD_570_ of PC for solids: 0.396 ± 0.098). In addition to these historical means, all relative tissue viability values <50 % were considered acceptable.
- AC for tissue variability*:* The relative inter-tissue variability (ITV%) between the two tissues of a treatment group was considered acceptable if it was ≤20 %.

#### BCOP assay

The BCOP assay was conducted according to OECD TG 437 [3].

##### Reagents and technical equipment, positive and negative controls

The following technical equipment was applied: Corneal holders (LAB Research, Hungary, or BASF SE, Germany), opacitometer (BASF-OP2.0, BASF SE, Germany), spectrophotometer (Sunrise™ Absorbance Reader, Tecan Group Ltd. Switzerland, measurement using filter wavelength of 490 nm without reference filter). The following reagents were used (all supplied by Biochrom AG, Germany, unless otherwise noted): Hanks’ Balanced Salt Solution (HBSS) containing 1 % (v/v) Penicillin/Streptomycin (10 000 IU/10 000 μg/mL); Eagle’s Minimum Essential Medium (MEM) without phenol red containing fetal calf serum and 1 % (v/v) Penicillin/Streptomycin; Eagle’s MEM with phenol red, sodium fluorescein (Merck KGaA, Germany) diluted in PBS.

Highly de-ionized water was used as NC. For the materials that were supplied as dry-powder test items, imidazole (CAS No. 288–32–4; Sigma Aldrich, Germany) 20 % (w/v) dissolved in highly de-ionized water was used as PC. For Ag NM-300 K, its dispersant, Ag NM-300 K DIS, and aSiO_2_-susp 1 % (w/v) sodium hydroxide (Sigma Aldrich, Germany) diluted with highly de-ionized water was used as PC.

##### Mounting of corneas and measurement of initial corneal opacity

Bovine corneas were mounted in corneal holders. Both the anterior and posterior chambers of these holders were filled to excess with pre-warmed Eagle’s MEM (without phenol red). After equilibration in a vertical position at 32 °C for at least 1 h, the medium in both chambers was replaced with fresh pre-warmed medium and the *initial corneal opacity* was measured.

##### Test material application, incubation and removal

Generally, each treatment group (NC, PC, or test material) consisted of 3 corneas. Before application of the test materials, the medium in the anterior chamber was removed.

- For the NC, the anterior chambers were filled with 750 μL highly de-ionized water and, for the PC, with 750 μL of the 20 % (w/v) imidazole solution.
- For all **TiO**_**2**_, **CeO**_**2**_ and **SiO**_**2**_ NMs as well as **ZnO NM-110**, 750 μL of the 20 % (w/v) test material preparation was applied directly to the epithelial surface of the cornea (i.e. using the ‘*open chamber method’*).
- For **Ag NM-300 K** and **Ag NM-300 K DIS**, and **aSiO**_**2**_ **-susp**, 750 μL of the undiluted test material dispersions were applied.
- For **ZnO NM-111** and **MWCNT NM-401** homogenous 20 % suspensions of the test material in water could not be prepared. Therefore 33 mg and 48 mg of undiluted dry-powder ZnO NM-111 and MWCNT NM-401, respectively, were applied with a sharp spoon, covering the entire corneas surface with these amounts.
- The volumes of **MWCNT NM-400 and NM-402** were too high to allow preparation of a w/v suspension. Therefore, 20 % (w/w) dry suspensions were prepared mixing 750 mg MWCNT NM-400 or NM-402 in highly de-ionized water shortly before application by stirring with a spatula.
- **Quartz dust DQ12, talc** and the three **organic pigments** Pigment Red 57:1, Pigment Yellow 95, and Pigment Black 32 were applied undiluted (120, 80, 48, 45, and 40 mg, respectively).

For all NMs that were delivered as **dry-powder test items** and **aSiO**_**2**_ **-susp**, the corneas were incubated in a horizontal position at 32 °C for 4 h as prescribed for non-surfactant solids in the OECD TG. **Ag NM-300 K**, and **Ag NM-300 K DIS** were tested with a 10 min application and 2-h post incubation protocol as prescribed for liquids.

Upon completion of the incubation period, the **NC**, **PC** and test materials were removed from the anterior chamber with a syringe, and the respective epithelia were washed at least 3 times with Eagle’s MEM (containing phenol red) and once with Eagle’s MEM (without phenol red). Both chambers were then refilled with fresh Eagle’s MEM (without phenol red). In the second test runs assessing **Ag NM-300 K** and **Ag NM-300 K DIS**, the glasses of the cornea holders were removed for easier test material removal. As assessed by IVIS and histopathological evaluation, this open chamber washing procedure was shown not to injure corneal tissue (data not shown).

##### Measurement of final corneal opacity and permeability and calculation of in vitro irritancy score

The *final corneal opacity* was measured, and the opacity change per cornea was calculated by subtracting the initial from the final opacity value. Subsequently, the mean opacity change of the NC was subtracted thereby providing the corrected opacity change. Test results were provided as means of all corrected opacity changes per treatment group.

To determine *corneal permeability*, the medium in the anterior chamber was replaced by 1 mL sodium fluorescein solution (5 mg/mL for solid test items; 4 mg/mL for liquid test items) and incubated in a horizontal position for 90 min at 32 °C. The amount of sodium fluorescein that permeated through the corneas was measured spectrophotometrically. Three aliquots per cornea were transferred to a 96-well microtitre plate and the optical density value (OD_490_) was determined subtracting the mean blank OD_490_ (blank = Eagle’s MEM without phenol red) from the OD_490_ of each cornea. Corrected OD_490_ values were calculated by subtracting the mean OD_490_ values of the corresponding NC. Final test results were calculated as means of all corrected OD_490_ values per treatment group.

The In Vitro *Irritancy Score (IVIS)* was calculated per treated cornea and finally the mean IVIS per treatment group ± standard deviation (SD) was determined: IVIS = mean opacity value + (15 x mean permeability value). An IVIS >55 indicates a risk of serious damage to the eyes.

##### Acceptance criteria

In case one of the following ACs laid down in OECD TG 437 was not met, repetition of the BCOP assay was considered.

- *AC for the NC:* The NC responses should be lower than the established upper limits for background opacity and permeability values for the respective NC.
- *AC for the PC:* The IVIS calculated for the PC should not lie outside the twofold range of the SDs of the historical mean (i.e. 88.0–147.1 for imidazole and 101.6–204.0 for sodium hydroxide).
- *AC for the treatment groups:* At least 2 of the 3 corneas per treatment group should provide predictions that coincide with the mean of all 3 corneas, and none of the corneas should provide a discordant prediction of 10 IVIS units above or below the cut-off threshold of 55.

##### Histopathological evaluation

For *histopathological evaluation* by light microscopy, the corneas were fixed in 10 % neutral buffered formalin for at least 24 h and trimmed along the whole diameter (2 stripes of 3–4 mm width). They were histotechnically processed with a standard method for light microscopy and stained with Hematoxylin and Eosin (Merck KGaA, Germany). Histopathological findings were assessed in the epithelium based on the depth of injury by using a standard semi-quantitative grading system (from 1 to 5) that is related to the extent of affected cell layers beginning from the corneal surface (squamous cell layer) down to the basal cell layer. This depth of the injury has been proposed as a predictor of the severity and reversibility of effects [41–43] and has been taken up in an OECD guidance document related to the BCOP assay [44]. In the stroma, tissue swelling and keratocyte changes were evaluated. These findings were summarized in a so-called Histopathological Score of Irritation (HSI) assigned for each cornea ranging from 0 = no irritation to IV = severe irritation (*cf.* Kolle et al*.* [45] for further details). HSI IV was assessed as ‘severe irritation’; HSI I, II and III were overall assessed as ‘non-severe irritation’; HSI 0 was regarded as no irritation.

## Results

### EpiOcular™-EIT

#### Fulfilment of the acceptance criteria

The **ACs for the NC and the PC** were always met in the EpiOcular™-EIT performed in accordance with either the protocol variants 1 and 2 (Additional file 1: Table SI-1). If **ACs for tissue variability** were not met in specific test runs, their repetition was considered (*cf.* Supplementary Information for further details).

#### Test results, variant 1

After washing, test material residues were observed on the EpiOcular™ tissues treated with the TiO_2_ or CeO_2_ NMs or MWCNT NM-401 or NM-402. However, since none of the test materials was able to reduce MTT directly, it was concluded that these residues do not to interfere with the MTT assay.

**Fig. 1:**
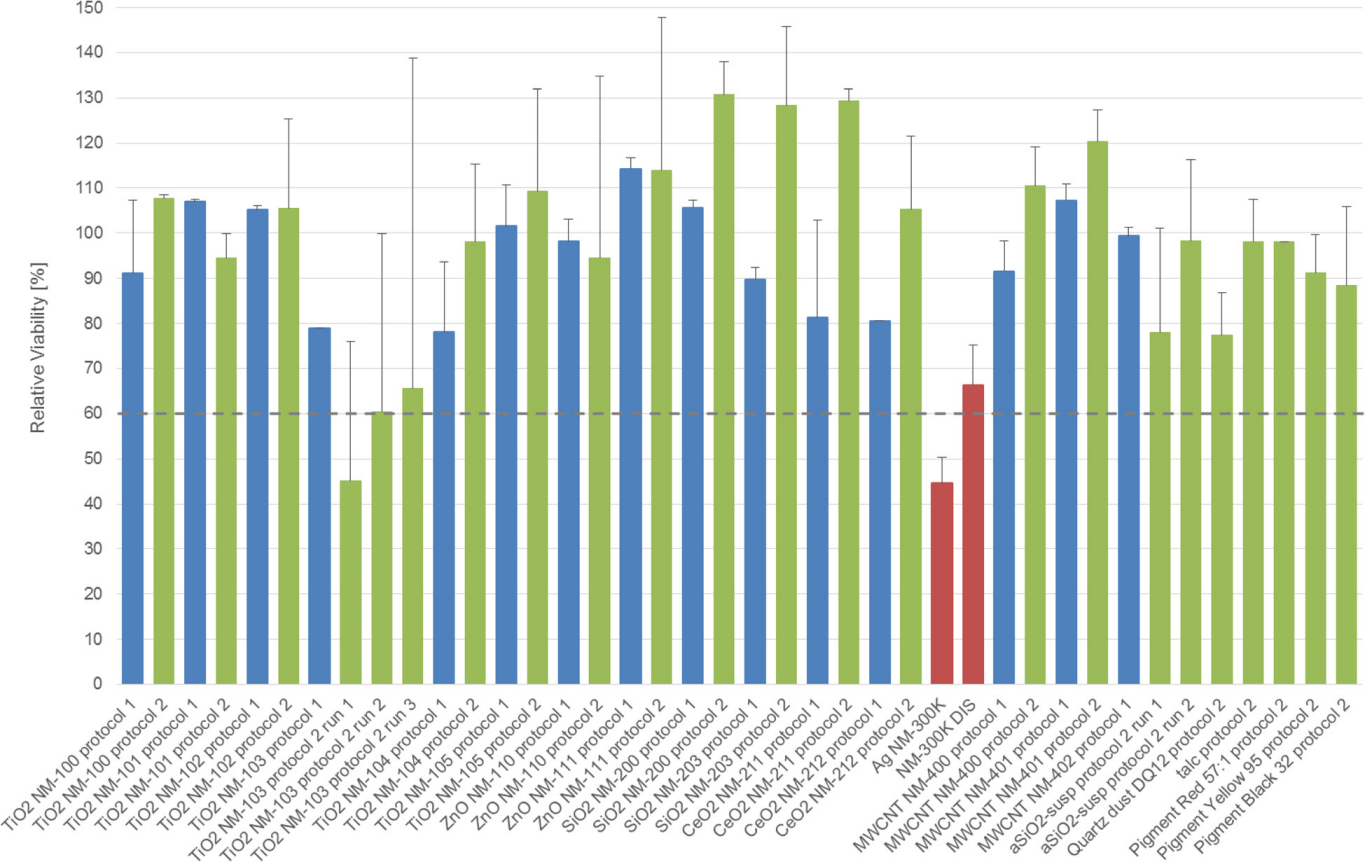
Mean relative tissue viability of two EpiOcular™ tissues plus inter-tissue difference obtained in the EpiOcular™-EIT. Blue bars represent results obtained applying the protocol for solids ‘variant 1’ (90 min exposure; 12 min post-exposure), green bars - protocol for solids ‘variant 2’ (6 h exposure; 25 min post-exposure) and red bars - protocol for liquids (30 min exposure; 12 min post-exposure)

For all **dry-powder NMs**, mean tissue viabilities above 60 % relative to the NC were calculated (Fig. 1 and Table 3; *cf.* Additional file 1: Table SI-1 for the corresponding NC and PC values). Hence, **none of the dry-powder NMs** revealed eye irritation potential in the EpiOcular™-EIT under the chosen test conditions (i.e. indicating likelihood of ‘neither Category 1 nor 2’).

Eye irritating potential was only recorded for **Ag NM-300 K** (individual relative tissue viabilities: 42 and 48 %; mean relative tissue viability: 45 %; i.e. all values ranged below the cut-off value of 60 % indicating likelihood of ‘either Category 1 or 2’). For the Ag dispersant **Ag NM-300 K DIS**, a mean relative tissue viability of 66 % and individual relative tissue viabilities of 62 and 71 % were recorded, i.e. all values ranged above the cut-off value of 60 %, thereby indicating likelihood of ‘Non-category’. Nevertheless, the mean relative tissue viability value recorded for Ag NM-300 K DIS was the second lowest value determined for any of the 17 OECD representative NMs and was just 1 % above the borderline range described for the EpiOcular™-EIT in OECD TG 492. Since the dispersed silver Ag NM-300 K elicited more pronounced effects than its dispersant alone, the effects induced by Ag NM-300 K in the EpiOcular™-EIT were assessed as being – at least partially – elicited by the silver particles (Table 3).

#### Test results, variant 2

After the 1^st^ test run conducted in accordance with the EpiOcular™-EIT variant 2, mean relative tissue viabilities >60 % (indicating likelihood of ‘neither Category 1 nor 2’) with concordant satisfactory ITV% were determined for **TiO**_**2**_ **NM-100, NM-101, NM-102, NM-104, NM-105, CeO**_**2**_ **NM-212, and MWCNT NM-400 and NM-401** (Fig. 1, Table 4 and Additional file 1: Table SI-2). Also for **aSiO**_**2**_ **-susp, quartz dust 12, the three organic pigments and talc** the mean relative tissue viabilities consistently exceeded 60 % (Table 5).

**TiO**_**2**_ **NM-103** was submitted to altogether three test runs. In the first two test runs, mean relative tissue viabilities of 45 and 60 % were recorded, however the ITV% exceeded 20 % (*cf.* 3.1.1). Therefore TiO_2_ NM-103 was submitted to a 3^rd^ test run. Here, slightly higher relative tissue viability was recorded (66 %), but again the ITV% exceeded 20 %. Therefore, the assessment of TiO_2_ NM-103 was terminated and its outcome was evaluated as inconclusive (Fig. 1 and Table 4).

Due to failure to meet the AC for tissue variability or high optical density values recorded in single measurements of the 1^st^ test run (*cf.* 3.1.1), two test runs each were conducted to evaluate the eye irritating potential of the other test materials: For the uncoated **ZnO NM-110**, the final mean relative tissue variability value (68 %) ranged just above the threshold value, whereas it attained 91 % for the coated **ZnO NM-111**. For **SiO**_**2**_ **NM-200** and **NM-203** and **CeO**_**2**_ **NM-211**, all individual and mean relative tissue variability values ranged above 90 % (apart from one individual value of 82 % recorded for SiO_2_ NM-203). Therefore, all of these findings were assessed as indicating likelihood of ‘neither Category 1 nor 2’ even though the ITV% exceeded 20 % in the 2^nd^ test runs of the SiO_2_ NMs (Table 4).

### BCOP assay

#### Fulfilment of the acceptance criteria

The **AC for the NC** was always met. Due to high opacity scores, one PC did not meet the **AC for the PC** (Additional file 1: Table SI-2). However, since all other ACs were met and the test material results were unambiguous, the study was assessed as being valid. In case the **AC for the treatment groups** was not met, repetition of the test run was considered (*cf.* Supplementary Information for further details).

#### NM agglomeration

As determined by AUC and / or LD as relevant (*cf.* test materials and particle characterisation), most dry-powder NMs suspended in water (i.e. as prepared for the BCOP assay) are predominantly present as agglomerates around 1 μm diameter. For MWCNT NM-400, agglomerate sizes even exceeded 10 μm. Dispersed fractions of the total dose with diameters up to 1 μm that exceeded 50 % of the total dose (i.e. indicating higher dispersibility) were recorded for TiO_2_ NM-100, NM-101, and NM-104, SiO_2_ NM-203 and CeO_2_ NM-212. Only SiO_2_ NM-203 prevailed in small agglomerates below 100 nm. Lower than 50 % of the total dose, but still noteworthy fractions of small agglomerates below 100 nm were further recorded for CeO_2_ NM-211 and TiO_2_ NM-100.

#### Test results, dry-powder test items

**Fig. 2:**
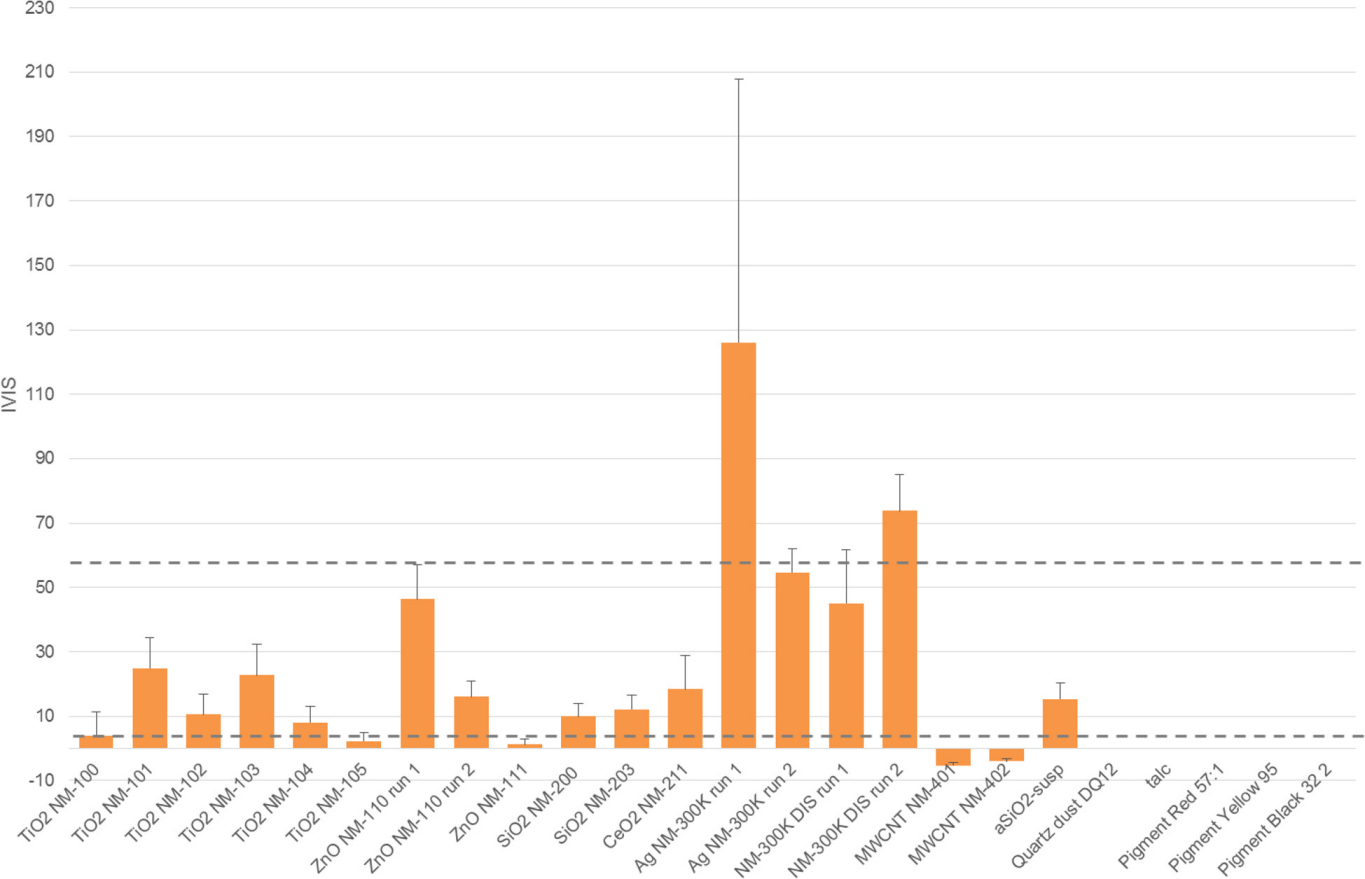
Mean BCOP assay IVIS (opacity + 15 x permeability) plus standard deviation. Abbreviations: IVIS: in vitro irritation score

None of the OECD representative **TiO**_**2**_, **SiO**_**2**_, or **CeO**_**2**_ **NMs** or **MWCNTs** induced serious eye damage in the BCOP as assessed by the respective IVIS that were far below the cut-off value of 55 (Fig. 2 and Table 6; *cf.* Additional file 1: Table SI-2 for the corresponding NC and PC values). Likewise, the **coated ZnO NM-111** did not induce serious eye damage in the BCOP (IVIS 0.01 ± 0.03). For the **uncoated ZnO NM-110**, a borderline result of 49.5 ± 10.7 was obtained in the 1^st^ test run (individual cornea IVISs 48.5, 60.7, 39.4). Therefore, a 2^nd^ test run was performed indicating no serious eye damage (mean IVIS 16.1 ± 4.7; individual cornea IVISs 15.3, 21.1, and 11.9).

**Table 6:** Mean opacity and permeability values and corresponding in vitro irritation score (IVIS) of 16 OECD representative nanomaterials (and the Ag NM dispersant) determined in the BCOP assay

Test material	TiO_2_ NM-100	TiO_2_ NM-101	TiO_2_ NM-102	TiO_2_ NM-103	TiO_2_ NM-104	TiO_2_ NM-105	ZnO NM-110 (1^st^ test run)	ZnO NM-110 (2^nd^ test run)	ZnO NM-111	SiO_2_ NM-200	SiO_2_ NM-203	CeO_2_ NM-211	CeO_2_ NM-212	Ag NM-300 K (1^st^ test run)	Ag NM-300 K (2^nd^ test run)	Ag dispersant NM-300 K DIS (1^st^ test run)	Ag dispersant NM-300 K DIS (2^nd^ test run ^a^)	MWCNT NM-400	MWCNT NM-401	MWCNT NM-402
Volume or mass applied	750 μL; 20 % (w/v)	750 μL; 20 % (w/v)	750 μL; 20 % (w/v)	750 μL; 20 % (w/v)	750 μL; 20 % (w/v)	750 μL; 20 % (w/v)	750 μL; 20 % (w/v)	750 μL; 20 % (w/v)	33 mg; undiluted	750 μL; 20 % (w/v)	750 μL; 20 % (w/v)	750 μL; 20 % (w/v)	750 μL; 20 % (w/v)	750 μL; undiluted	750 μL; undiluted	750 μL; undiluted	750 μL; undiluted	750 mg; 20 % (w/w)	48 mg. undiluted	750 mg; 20 % (w/w)
Mean opacity value ± SD	4.2 ± 7.2	24.7 ± 4.8	10.7 ± 6.1	23.8 ± 6.8	10.7 ± 4.3	2.2 ± 2.8	46.6 ± 9.1	15.0 ± 3.0	1.1 ± 1.1	9.7 ± 3.4	11.8 ± 3.7	18.0 ± 9.9	17.2 ± 5.4	123.9 ± 80.6	2.8 ± 0.6	43.9 ± 16.3	66.6 ± 2.9	−4.7 ± 2.2	−5.4 ± 0.7	−3.8 ± 0.8
Mean permeability value ± SD	−0.01 ± 0.00	0.22 ± 0.31	−0.12 ± 0.01	0.14 ± 0.18	0.04 ± 0.04	0.00 ± 0.00	0.20 ± 0.10	0.08 ± 0.12	0.01 ± 0.03	0.02 ± 0.05	0.03 ± 0.05	0.03 ± 0.04	−0.02 ± 0.01	0.14 ± 0.08	3.46 ± 0.46	0.08 ± 0.03	0.48 ± 0.56	−0.01 ± 0.00	0.01 ± 0.03	−0.01 ± 0.01
IVIS ± SD	4.1 ± 7.1	28.0 ± 4.2	10.6 ± 6.2	25.9 ± 5.7	11.2 ± 4.0	2.2 ± 2.8	49.5 ± 10.7	16.1 ± 4.7	1.2 ± 0.7	10.0 ± 4.0	12.2 ± 2.9	18.5 ± 10.5	16.9 ± 5.3	126.0 ± 81.8	54.7 ± 6.3	45.0 ± 16.7	73.7 ± 11.3	−4.8 ± 2.3	−5.3 ± 0.4	−3.9 ± 0.7

Ag NM-300 K and Ag NM-300 K DIS were tested using the BCOP treatment protocol for liquids (10 min test substance exposure, 2 h post-exposure incubation) while all others were tested according to the treatment protocol for solids (4 h test substance exposure without further post-incubation)

Abbreviations: *IVIS* In vitro irritation score, *NC* negative control, *PC* positive control, *SD* standard deviation

^a^ Only two corneas were treated, since the available test substance was not sufficient to treat a third cornea

Likewise, **quartz dust DQ 12, the three organic pigments and talc** did not induce serious eye damage in the BCOP, with all IVIS being 0 (Table 7).

**Table 7:** Mean opacity and permeability values and corresponding in vitro irritation score (IVIS) of aSiO_2_-susp, quartz dust DQ12, talc, and three organic pigments determined in the BCOP assay

Test material	aSiO_2_-susp	Quartz dust DQ12	Pigment Red 57:1	Pigment Yellow 95	Pigment Black 32	Talc
Volume or mass applied	750 μL	120 mg	48 mg	45 mg	40 mg	80 mg
Mean opacity value ± SD	14.9 ± 4.7	0.0 ± 0.0	0.0 ± 0.0	0.0 ± 0.0	0.0 ± 0.0	0.0 ± 0.0
Mean permeability value ± SD	0.04 ± 0.02	0.0 ± 0.0	0.0 ± 0.0	0.0 ± 0.0	0.0 ± 0.0	0.0 ± 0.0
IVIS ± SD	15.4 ± 4.5	0.0 ± 0.0	0.0 ± 0.0	0.0 ± 0.0	0.0 ± 0.0	0.0 ± 0.0

All substances were applied undiluted using the BCOP treatment protocol for solids (4 h test substance exposure without further post-incubation)

Abbreviations: *IVIS* In vitro irritation score, *NC* negative control, *PC* positive control, *SD* standard deviation

**Fig. 3:**
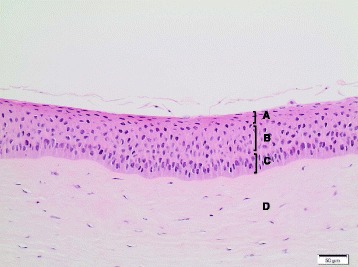
Bovine cornea treated with negative control deionized water (10x, HE stain); HSI: 0. **A** Squamous cell layer; **B** wing cell layer; **C** Basal cell layer; **D** Stroma. Abbreviations: HE: Hematoxylin and Eosin; HSI: Histological score of irritation

**Fig. 4:**
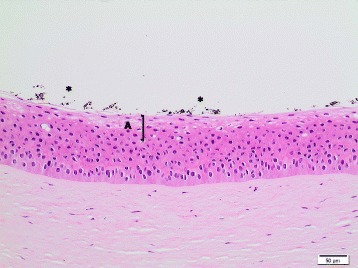
Bovine cornea treated with CeO_2_ NM-211 (10x, HE stain); HSI: I. **A** Multifocal vacuolation in the squamous and upper wing cell layers; multifocal desquamation and brown particle residues (*) on epithelial surface. Abbreviations: HE: Hematoxylin and Eosin; HSI: Histological score of irritation

Generally, no *histopathological findings* were observed for the corneas treated with the **dry-powder NMs**. Minimal findings (mostly minimal multifocal or diffuse desquamation) were only observed for **TiO**_**2**_ **NM-101**, **ZnO NM-110**, **CeO**_**2**_ **NM-211** and **NM-212**, and for these test materials an HSI of I (minimal) was assigned (Table 8). Of note, for these four NMs, IVIS >15 were calculated, whereas all dry-powder NMs without histopathological findings had IVIS ≤15. Figure 3 presents the histopathological image of a bovine cornea treated with the negative control deionized water, and Fig. 4 the one of a cornea exposed to CeO_2_ NM-211. For **quartz dust DQ12**, one cornea remained without findings, whereas minimal findings (multifocal desquamation) were recorded for two corneas (Table 8).

**Table 8:** Histopathological evaluation of the bovine corneas incubated with 16 OECD representative nanomaterials or the Ag dispersant

Test material	Test run	HSI	Histopathological findings
TiO_2_ NM-100	1	0	Multifocal brown pigment remnants on squamous surface
TiO_2_ NM-101	1	I	Multifocal desquamation and brown granules on the epithelial surface
TiO_2_ NM-102	1	0	Brown granules on squamous surface
TiO_2_ NM-103	1	0	No findings
TiO_2_ NM-104	1	0	No findings
TiO_2_ NM-105	1	0	No findings
ZnO NM-110	1	I	Multifocal desquamation
ZnO NM-110	2	I	Diffuse desquamation
ZnO NM-111	1	0	No findings
SiO_2_ NM-200	1	0	No findings
SiO_2_ NM-203	1	0	No findings
CeO_2_ NM-211	1	I	Multifocal vacuolation in the squamous and wing cell layers; multifocal desquamation and brown granules on the epithelial surface
CeO_2_ NM-212	1	I	Multifocal desquamation and brown granules on the epithelial surface
Ag NM-300 K	1	IV	All corneas: diffuse cell loss in the squamous and wing layers; two corneas, additionally: multifocal eosinophilic precipitate on the basal lamina; multifocal cell lysis in the basal layer; pyknosis of the upper keratocytes of the stroma
Ag NM-300 K	2	n.e.	Corneal epithelium completely detached and missing
Ag dispersant NM-300 K DIS	1	II	Diffuse vacuolation / edema of the squamous and upper wing cell layers; desquamation of the squamous cell layer
Ag dispersant NM-300 K DIS	2	IV	Multifocal vacuolation of basal layer, epithelial detachment from basal lamina, multifocal eosinophilic precipitate between basal layer and stroma
MWCNT NM-400	1	0	No findings
MWCNT NM-401	1	0	No findings
MWCNT NM-402	1	0	No findings
aSiO_2_-susp	1	II	Diffuse cell loss
Quartz dust DQ12	1	0-I	1 cornea: no findings; 2 corneas: multifocal desquamation
Pigment Red 57:1	1	0	No findings
Pigment Yellow 95	1	0	No findings
Pigment Black 32	1	0	No findings
Talc	1	0	No findings

*HSI* histopathological score of irritation (HSI) of 0 = no findings, *I* = minimal, *II* = mild, *III* = moderate, *IV* = severe

*n.e* = evaluation not possible due to technical artefacts

#### Test results, liquid test items

**Fig. 5:**
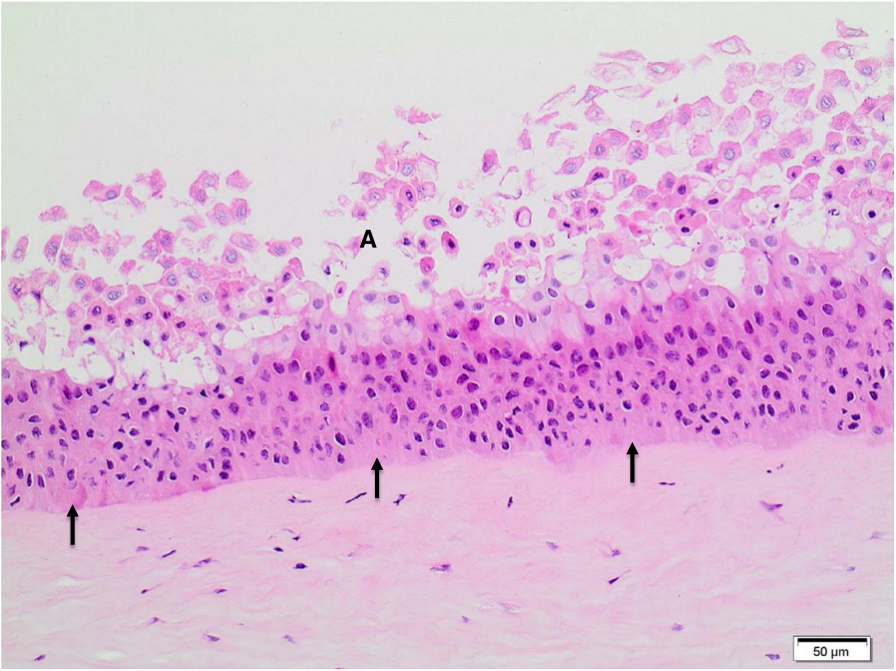
Bovine cornea treated with Ag NM-300 K (10x, HE stain); HSI: IV. **A** Diffuse cell loss of squamous and wing cell layers;: Multifocal karyolysis in the basal cell layer (**↑**). Abbreviations: HE: Hematoxylin and Eosin; HSI: Histological score of irritation

**Fig. 6:**
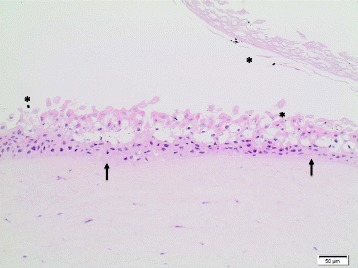
Bovine cornea treated with Ag NM-300 K (10x, HE stain); HSI: IV. Diffuse cell loss of squamous and wing cell layers; Multifocal karyolysis in the basal cell layer (↑) and Ag NM-300 K particle residues (*). Abbreviations: HE: Hematoxylin and Eosin; HSI: Histological score of irritation

For **Ag NM-300 K**, varying amounts of the test material remained on the cornea after the washing procedure of the 1^st^ test run causing dark-brown patches on the cornea surface. Histological evaluation of these corneas indicated mild (one cornea) or severe (two corneas) eye irritation with an overall HSI of IV (Table 8 and Figs. 5 and 6). Due to the high variability between the individual corneas of the 1^st^ test run (individual cornea IVISs of 117.0, 212.0, and 49.1), a 2^nd^ test run was performed using the open chamber washing procedure to remove residual test material from the cornea surfaces. The mean IVIS of the second run was 54.7 ± 6.3 (with individual cornea IVISs of 62.0, 50.8 and 51.3). Even though no residues were observed in the 2^nd^ test run, histopathological evaluation revealed that the corneal epithelium was completely detached and missing. Since such a finding was not observed in the 1^st^ test run, it was attributed to the intensified washing procedure and no HSI was assigned since it was possible not differentiate whether the epithelium was missing as a test material-related effect and/or due to a putative washing artefact. Concordantly, the permeability measurements produced low values in the 1^st^ test run, but very high values in the 2^nd^ test run (0.14 ± 0.08 versus 3.46 ± 0.46; Table 6). Based upon these observations, the BCOP assay, as performed in the present study, does not appear suitable to assess the eye damaging potential of Ag NM-300 K.

**Fig. 7:**
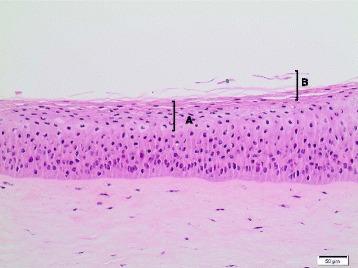
Bovine cornea treated with Ag NM-300 K DIS (10x, HE stain); HSI: II. **A** Diffuse edema / vacuolation of the squamous and upper wing cell layers; **B** desquamation of the squamous cell layer. Abbreviations: HE: Hematoxylin and Eosin; HSI: Histological score of irritation

**Fig. 8:**
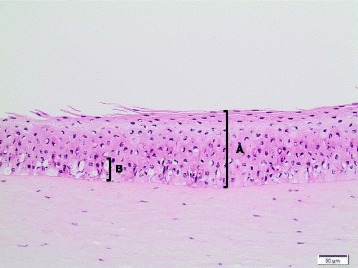
Bovine cornea treated with Ag NM-300 K DIS (10x, HE stain); HSI: IV. **A** Vacuolation of all epithelial cell layers, accentuated in the basal cell layer **B** Abbreviations: HE: Hematoxylin and Eosin; HSI: Histological score of irritation

Also for the Ag dispersant **Ag NM-300 K DIS**, high variability between the individual corneas used in the 1^st^ test run was recorded (mean IVIS: 45.0 ± 16.7 with individual cornea IVISs of 60.1, 47.9, and 27.1), and a 2^nd^ test run was performed using the open chamber washing procedure (albeit only with 2 corneas since the available test material was not sufficient to treat a third cornea; with individual IVIS scores of 65.7 and 81.7, respectively). Accordingly, of the total of five corneas tested, two produced IVIS below the cut-off for serious eye damage and three corneas produced values above this value. The overall mean IVIS for both test runs was calculated to be 56.5. Hence, it ranged just above the cut-off value of 55 indicating a potential for serious or irreversible eye damage, however very close to the cut-off and with a rather high SD of 20.5. Due to this high inter-cornea variability, the borderline positive BCOP result for Ag NM-300 K DIS was assessed as inconclusive. Histological evaluation (Table 8) revealed changes indicating mild (1^st^ test run; HSI: II; Fig. 7) or severe eye irritation (2^nd^ test run; HSI: IV; Fig. 8).

For **aSiO**_**2**_ **-susp**, the IVIS was determined to be 15.4 (i.e. far below the cut-off value of 55; Table 7), and mild findings (diffuse cell loss; HSI II) were observed in the histopathological evaluation (Table 8).

### Two-tier EpiOcular™ - BCOP eye irritation testing strategy

According to the results of the EpiOcular™-EIT, **all dry-powder test items and aSiO**_**2**_ **-susp** would be classified as ‘neither Category 1 nor 2’ substances, i.e. as having no eye irritating potential. By contrast, **Ag NM-300 K** would be classified as ‘Category 1 or 2’, i.e. as having the potential to either induce serious, irreversible or reversible eye damage. Further according to the results of the EpiOcular™-EIT, the Ag dispersant **Ag NM-300 K DIS** would be classified as ‘Non-category’ substance even though its relative tissue viability score (66 %) lay close to the cut-off value of 60 %.

In agreement with the results obtained for the dry-powder test items and aSiO_2_-susp in the EpiOcular™-EIT, also in the BCOP assay, **the metal and metalloid oxide NMs (ZnO, TiO**_**2**_ **, CeO**_**2**_ **, SiO**_**2**_ **) and the MWCNTs** were assessed as not inducing severe eye damage, i.e. as being ‘Not category 1’.

**Ag NM-300 K** was assessed as ‘Category 1 or 2’ in the EpiOcular™-EIT. Accordingly, in a 2-tiered bottom-up testing strategy the results from the BCOP assay should be used to distinguish ‘Category 1’ from ‘Category 2’ substances [25]. The results of the BCOP assay for Ag NM-300 K, however, did not allow a definite evaluation. In the BCOP assay, the borderline positive outcome for the silver dispersant **Ag NM-300 K DIS**, ranging just above the cut-off value indicating severe irritation, remains inconclusive due to high inter-cornea variability. Taking into account that Ag NM-300 K DIS also elicited an effect (albeit negative) close to the cut-off value in the EpiOcular™-EIT, the eye damaging potential of this liquid surfactant could not be ruled out, but also could not be assessed with certainty in the present study.

Interestingly, whereas none of the silicon dioxide test materials revealed eye irritating potential in either the EpiOcular™-EIT or the BCOP assay, during histopathological evaluation only the two dry powder test items amorphous precipitated SiO_2_ NM-200 and amorphous pyrogenic SiO_2_ NM-203 remained without findings, whereas a HSI of 0-I was recorded for the dry powder crystalline quartz dust DQ12 and a HSI of II for the amorphous aSiO_2_-susp that was provided as 40 % suspension.

## Discussion

### Relevance of study results for hazard assessment

A broad panel of inorganic NMs covering one metal NM (Ag), different metal oxides (anatase, rutile and anatase-rutile TiO_2_, coated and uncoated ZnO, uncoated CeO_2_), three amorphous SiO_2_ (two supplied as powders and one as suspension), and three MWCNTs, three organic pigments, as well as non-nanosized quartz dust and talc were submitted to in vitro eye irritation studies. Selecting test materials from the set of OECD representative NMs aimed at facilitating comparability of the test results to studies performed by other research groups. Taking into account that none of the dry-powder test items (i.e. all test materials, except for Ag NM-300 K, Ag NM-300 K DIS, and aSiO_2_-susp) elicited eye irritation in either the EpiOcular™-EIT (using 2 protocol variants) or the BCOP assay including histopathological evaluation, the following paragraphs discuss the relevance of this outcome for human eye irritation assessment.

The EpiOcular™-EIT has been reported to have an overall low false negative rate of 4 % [4]. By contrast, a high false negative rate specifically for solids has been recorded for the BCOP assay (that contributes to the overall false negative rate of 14 % in determining ‘Category 1’ substances [3]). However, insoluble (solid) test items are generally difficult to apply in many in vitro test systems, and they may also lead to variable exposure conditions in vivo, for instance by causing mechanical corneal irritation in addition to toxicological effects [3, 46].

The results from the BCOP assay were only evaluated to determine whether test materials might cause severe or irreversible eye damage as determined if the IVIS exceeds the threshold value of 55. In accordance with the revised OECD TG 437 [3], additionally, an IVIS ≤3 indicates that the test material does not cause eye irritation or corrosion and may be assessed as ‘Non-category’. In the present study, IVIS ≤3 were obtained for TiO_2_ NM-105 and coated ZnO NM-111, but not for the corresponding non-coated ZnO NM-110 for which a borderline IVIS of 49.5 was determined in the 1^st^ test run and an IVIS of 16.1 in the 2^nd^ test run. This may indicate that material coating or surface functionalization may mitigate unwanted effects. In a rat short-term inhalation study, the histopathological effects caused by coated ZnO NM-111 in the rat lung were less severe or had a lower incidence than those elicited by micron-scale uncoated ZnO [7] and in rat precision-cut lung slices ZnO NM-110 elicited more pronounced cytotoxicity than ZnO NM-111 [38]. Additionally, IVIS ≤3 were recorded for quartz dust DQ12, the three organic pigments, and talc.

Ag NM-300 K was supplied in suspension and the corresponding dispersant (Ag NM-300 K DIS) without Ag was also evaluated. In the EpiOcular™-EIT, Ag NM-300 K was assessed as ‘Category 1 or 2’. When applying this test material in the BCOP assay, dark-brown patches remained on the surface of the treated corneas (unless the washing procedure was intensified, which however resulted in detachment of the corneal epithelium). In vivo, discolouration of the cornea that is not reversible within 21 days would result in the test material’s classification as ‘eye irritant’. However, for Ag NM-300 K, in vivo data are unavailable. Therefore, the in vivo relevance of the outcome of the BCOP assay remains unclear. Recently, Plodikova et al. reported how different modifications of the rinsing procedure within the BCOP assay may enhance the removal efficiency of highly viscous and coloured samples. Depending on the selected rinsing procedure, the calculated IVIS values were significantly altered, mainly due to altered opacity scores, whereas the permeability values mostly remained unaffected [47].

The outcomes of both the EpiOcular™-EIT and BCOP assay were negative for the second NM that was supplied in suspension, i.e. aSiO_2_-susp, and a HSI of II was recorded upon histopathological evaluation of the corneas treated with aSiO_2_-susp. Accordingly, for all three liquid (suspended) test items, higher HSI of II-IV were recorded than for the dry-powder test items (HSI of 0-I).

### Comparison of in vitro and in vivo eye irritation

To elucidate the relevance of the outcomes of the BCOP assay and the EpiOcularTM-EIT for hazard assessment, in the following, they are compared to the outcomes of in vitro or in vivo eye irritation studies investigating other TiO_2_, ZnO, amorphous SiO_2_, CeO_2_, Ag, MWCNTs, or organic pigments. For the organic pigments, the existing in vivo eye irritation studies had been performed with the same sample in case of Pigment Black 32 or with samples of comparable grades. Therefore, direct in vitro-in vivo comparisons are possible for these test materials. For the other test materials, studies assessing non-nanosized counterparts were also taken into consideration, such as the key studies recorded in the corresponding REACH dossiers (www.echa.europa.eu/information-on-chemicals; accessed 7 April 2016).

In this respect, it should further be noted that the present data sets are not a subject for statistical analysis. Neither OECD TG 492 nor 437 require statistical analysis. While Cooper statistics are beneficial to analyze the predictive capacity of a new method, it was not applied it since the majority of in vitro-in vivo comparisons were only based upon assessments of comparable materials of the same chemical composition. Further, no dose-/concentration response curves could be recorded so that statistical analyses that are founded on such curves could also not be performed.

To date, there are no provisions in EC regulation 1907/2006 (REACH [1]) referring specifically to NMs. The legislative text deals with substances, as such, in whatever size, shape or physical state. Since substances at the nanoscale are substances in a specific form, they are included in the general registration dossier [1, 48]. Accordingly, NMs for which a bulk counterpart exists are registered together with this counterpart. The EU Commission [49] advised that NMs should be classified on a case-by-case basis giving due consideration to relevant available data on, e.g., the bulk form and read-across to other NMs.

Against this background, the eye irritancy categories assigned to the test materials as an outcome of the present study were compared to eye irritancy classifications from the REACH registration dossiers for titanium dioxide, zinc oxide, silica (covering all crystalline and amorphous SiO_2_), cerium dioxide, silver, and a specific MWCNT. The industrial mineral talc is exempt from REACH registration.

In all of these REACH dossiers, the key studies for eye irritation were in vivo tests performed in accordance with OECD TG 405. Mostly, rabbits were used, with the exception of the first key study for Ag (which corresponds to Maneewattanapinyo et al*.* [21]), which was performed using guinea pigs. For TiO_2_, SiO_2_, and Ag, key studies for eye irritancy assessment were conducted with NMs, the tested MWCNTs are NMs, as such, and organic pigments fall under the EU recommendation for the definition of NMs ([24] but are exempt from certain national NM-specific legislation). In the REACH dossier for ZnO, two supporting studies correspond to the EpiOcular™-EIT and BCOP assessments for ZnO NM-110 of the present study. In none of the mentioned REACH dossiers, eye irritancy Categories 1 or 2 were assigned (Table 1).

Hence, the eye irritancy assessments of the mentioned REACH dossiers back up the lack of effects observed for the NMs that were supplied as powder in the EpiOcular™– BCOP eye irritation testing strategy. Also for the organic pigments, mild transient effects clearly below the threshold for classification and labelling were recorded in the available in-house in vivo studies which is consistent with the outcome of the BCOP assay and EpiOcular™-EIT.

Similarly, in its *Opinion on nanoform TiO*_*2*_, the EU Commission’s Scientific Committee on Consumer Safety (SCCS; [50]) comes to the conclusion that the eye irritation potential of TiO_2_ NMs appears to be low. However, the SCCS cautions that this assessment is predominantly based upon two in vivo eye irritation studies assessing 15 % anatase / 85 % rutile TiO_2_ coated with trimethoxy-n-octyl-silane. In its *Opinion on nanoform ZnO*, the SCCS [51] assessed an uncoated ZnO NM (20 nm; dosage: 0.1 g of neat test material or 0.1 mL of a 25 % solution in olive oil) as being a ‘mild irritant’, also basing this conclusion on an in vivo rabbit eye irritation study. An extensive evaluation of the toxicological profile of nanosized and non-nanosized ZnO found the effects elicited by either material to be very similar [52].

In the REACH dossier for silver, two key studies for eye irritation were conducted with Ag NM suspensions (one of which an aqueous solution, the other one not further specified in the key study) and one key study with an Ag NM powder that was applied directly into the conjunctival sac of the rabbit eyes. Whereas no ocular effects were observed upon treatment with the Ag NM powder, the Ag NM suspensions caused minimal and fully reversible conjunctival redness in single animals only. All three key studies came to the conclusion that the tested Ag NMs were not irritating. Based upon the outcome of the 2-tier in vitro testing strategy applied in the present study, the surfactant-dispersed Ag NM-300 K preparation appears to elicit more pronounced eye irritation than Ag NMs that are supplied as powders or in aqueous suspension, and dark-brown patches remained on the surface of the corneas treated with Ag NM-300 K. In its *Opinion on nanosilver*, the EU Commission’s Scientific Committee on Emerging and Newly Identified Risks [53] highlighted a permanent bluish-grey discolouration of the skin or eyes as being the predominant adverse effects elicited by chronic human exposure to silver, as was also reported by Chiou [22]. Additionally, exposure to soluble silver compounds was reported to possibly produce liver and kidney damage, irritation of the eyes, skin, respiratory, and intestinal tract, and changes in blood cells [53].

In regard to available in vitro studies addressing the eye irritancy of NMs intended for non-medicinal uses, Sanders et al*.* exposed human ARPE-19 retinal pigment epithelial cells to 0.3-100 μg/ml of differently sized anatase, rutile and anatase/rutile TiO_2_ NMs. After 24-h test material exposure in the live/dead calcein-AM and propidium iodide assay with subsequent 4-h exposure continuation under UV radiation, the smaller TiO_2_ NMs (primary particle size (PPS): 25 and 31 nm; Transmission Electron Microscopy (TEM)) elicited the most pronounced phototoxicity (LC_50_ < 5 μg/ml), whereas the largest TiO_2_ NMs (PPS: 142 nm and 214 nm; TEM) were the least toxic [54]. In cultured eye-associated cells, Ag NMs supplied as aqueous suspensions did not affect cell viability: In human HCE-T corneal epithelial cells or eye-associated murine RAW264.7 macrophages, 2-6 μM Ag NMs/mL (PPS: 40 nm; 10 ppm Ag in the colloids) did not significantly affect cell viability, as assessed by cell counts and adenylate kinase release from damaged cells, nor did they induce different interleukins [55]. Evidently, the test systems and endpoint detection methods selected in the present study differ from those applied in the studies by Sanders et al*.* [54] and Santoro et al*.* [55] thereby impeding comparability of the test results. In rat precision-cut lung slices, Ag NM-300 K elicited pronounced tissue destruction, at effective in vitro dosages of up to 12 μg/cm^2^ cell culture surface area [38].

### Mechanisms of nanomaterial eye irritation

Increasingly, it is being recognized that nanoscale particle size alone does not dictate toxicity, but that intrinsic and system-dependent (i.e. test system-, matrix- or environment-dependent) material properties, such as chemical composition, solubility, and dispersibility, are more relevant determinants of NM toxicity [7, 8, 56, 57]. These material properties differ considerably for the 16 OECD representative NMs (*cf.* also Tables 1 and 2): The metal NM Ag NM-300 K and the uncoated and coated metal oxides ZnO NM-110 and NM-111, respectively, are soluble in water or biological media. Due to their chemical composition, these materials, upon dissolution, may shed toxic ions. By comparison, TiO_2_ and CeO_2_ NMs are poorly soluble, and the amorphous SiO_2_ NM-200 and NM-203 are partly soluble. For these metal oxide and metalloid oxides, the toxic potential, e.g. upon inhalation, is strongly affected by their respective biopersistence. For the MWCNTs, just as other fibres, a high aspect ratio combined with fibre rigidity and biopersistence accounts for toxic potential [57]. Of note, according to the ‘exclusion rules for eye irritation’ from the *German Federal Institute for Risk Assessment*, substances of very high molecular weight (>650 g/mol) and of very poor solubility in water and lipids are non irritating to the eye [58].

Taking into account their intrinsic and system-dependent material properties, knowledge on the mechanisms of toxicity of NMs is increasingly becoming available, albeit predominantly for the inhalation route of exposure. For Ag and ZnO NMs, toxicity appears mainly determined by the potential to shed toxic ions. These materials frequently elicit pronounced cytotoxicity in vitro, and in rats ZnO NMs have been observed to cause necrosis of the olfactory epithelium, the primary site of contact upon inhalation [7, 8]. TiO_2_ and CeO_2_ NMs may elicit the production of reactive oxygen species and inflammatory reactions; amorphous SiO_2_ has been observed to elicit membrane damage and lysis, and MWCNTs may cause fibre-related toxic effects [8, 59].

Against this background, it remains to be determined whether the mechanisms of toxicity (and underlying key material properties) that are relevant for the inhalation exposure route might also be determinants of effects upon topical exposure to the eye. For eye irritation, four different known modes-of-action have been described, i.e. (I) cell membrane lysis (breakdown of membrane integrity as might be elicited by, e.g., surfactants; such as the Ag NM-300 K dispersant), (II) saponification (breakdown of lipids by alkaline action), (III) coagulation (precipitation/denaturation of macromolecules), and (IV) actions on cellular macromolecules [44].

To account for the underlying biological processes that ultimately result in eye irritation classifications [60], it has been requested to incorporate mechanistic information into in vitro eye irritation testing strategies. For instance, the reversibility of effects may be reflected by corneal repair and recovery mechanisms (for reversibility of effects), such as renewal of the superficial lining of the corneal epithelium [33]. In an in vitro wound healing array, in which human corneal fibroblasts or human corneal epithelial cells were cultured to confluence with subsequent production of a ‘wound’ and imaging of the healing process and cell migration, ZnO NMs (PPS: 40-100 nm; hydrodynamic diameter in DMEM-F12 supplemented with 10 % fetal calf serum (DLS): 162 nm) were observed to impede both cell viability and wound healing [61]. These effects occurred at 15.8 μg/mL in the epithelial monolayers and at 45.2 μg/mL and above in the fibroblast cultures. By contrast, up to 108 μg/mL amorphous SiO_2_, and TiO_2_ (PPS: 55 nm, and 25 nm, respectively; hydrodynamic diameter: 172 nm, and 838 nm, respectively) elicited no such effects on the corneal cells [61]. Monodisperse SiO_2_ NMs (spherical (TEM); 40 nm (DLS); no further particle characterisation), however, applied in 1 mg/mL aqueous suspensions, were found to penetrate across isolated human corneal buttons [62].

As described in the OECD guidance document [44], the BCOP assay conducted in accordance with OECD TG 437 addresses the first three modes-of-action recognized for eye irritation (i.e. cell membrane lysis, saponification, and coagulation), and the fourth one (actions on cellular macromolecules) might also be addressed if histopathological information is available. In the present study, histopathological evaluation of the corneas submitted to the BCOP assay was performed to corroborate the outcome of this assay and potentially to contribute to defining mechanisms of eye irritancy of the tested NMs. In an earlier study investigating a broad spectrum of non-nanosized materials in the BCOP assay, histopathological assessment of the corneas corrected the classification of some false negatives, but also increased the overall number of false positives [26].

Since none of the dry-powder NMs elicited eye irritation in either the EpiOcular™-EIT or the BCOP assay in the present study, and the corresponding histopathological evaluation of the treated corneas resulted in very low HSI of 0 or I, the results of the current study do not provide information on different modes of eye irritating action for dry-powder NMs. Presumably, they are simply not eye irritating.

The surfactant Ag NM-300 K DIS, either alone or as dispersant in Ag NM-300 K most likely elicited the first mode-of-action for eye irritation, i.e. cell membrane lysis. Of note, the Ag NM-300 K DIS component Tween 20 is non-irritant both in vivo and in vitro [3]. It is currently unclear whether effects caused by Ag NM-300 K in the EpiOcular™-EIT and BCOP assay are – at least partially – attributable to the shedding of toxic ions. Likewise, it is unclear if the discolouration of the cornea would also be observable in vivo, and if so, if it would persist throughout 21-days post-administration resulting in classification of the material.

### Applicability of the EpiOcular™ - BCOP eye irritation testing strategy for the testing of nanomaterials

According to the results of the EpiOcular™-EIT, all dry-powder test items and aSiO_2_-susp would be classified as having no eye irritating potential. In agreement with this outcome, all of these test materials were assessed as not inducing severe eye damage, i.e. as being ‘Not category 1’ in the BCOP assay. Hence, for dry-powder NMs, the results of the present study point to the overall low eye irritating potential of these test materials.

Regarding the observations made for Ag NM-300 K and its dispersant Ag NM-300 K DIS, both in the EpiOcular™-EIT and the BCOP assay, further research is necessary to determine if the silver nanoparticles may cause eye irritating effects, either alone or in combination with the dispersants. The difficulties encountered when testing Ag NM-300 K and Ag NM-300 K DIS in the BCOP assay might not be specific to NMs, but may be similar to the ones commonly occurring when testing non-nanosized liquid and surfactant materials. Since oftentimes it is exactly such specific preparations of NMs that end up being handled either in occupational or consumer-related contexts, hazard assessment of the specific NM preparation is relevant for safety measures.

The EpiOcular™ – BCOP eye irritation testing strategy, as it was applied in the present study to assess a broad spectrum of NMs, follows the recommendations of the GHS [6] and the supplement to the updated OECD TG 405 on in vivo acute eye irritation and corrosion testing [2]. This supplement proposes a sequential testing strategy for eye irritation and corrosion that predominantly makes use of in vitro or *ex vivo* assays restricting in vivo testing to materials eliciting equivocal results in the non-animal test tiers. General applicability of the 2-tier testing strategy to distinguish ‘Category 1’ from ‘Category 2’ eye irritancy of NMs cannot be assessed by the outcome of the present study since only one material (i.e. Ag NM-300 K) was assessed as ‘Category 1 or 2’ in the EpiOcular™-EIT. As long as comprehensive data for NM in vitro and in vivo eye irritation potential covering the different relevant mechanisms of toxicity are unavailable, the applicability of the EpiOcular™ – BCOP eye irritation testing strategy to determine if a NM should be classified as either ‘Category 1’, ‘Category 2’ or ‘non-Category’ cannot be assessed with certainty. Nevertheless, the consistently negative findings obtained for the dry-powder NMs in both in vitro assays stand in accordance with eye irritation classifications in corresponding REACH registration dossiers.

A number of specificities have to be taken into account for in vitro testing of NMs, and these specificities also point to aspects of the OECD TGs that might require modification for NM testing as cautioned by the OECD WPMN [36]. For instance, the agglomerating properties of NMs in suspensions have to be addressed in the course of in vitro testing. The extent of NM agglomeration may affect its diffusion and sedimentation properties which in return may affect the proportion of the applied dose that reaches the in vitro test system within the given exposure duration [8, 37, 63, 64]. The differences between in vitro and in vivo effective dosages of identical test material preparations are expected to be much less pronounced in the context of local toxicity testing, than the differences recorded for inhalative or systemic effects. For local toxicity testing, NMs are directly topically applied to the 3D-test systems rather than suspended in cell culture media as required for most cellular assays. In the EpiOcular™-EIT, the test materials are applied undiluted. This may be an advantage for NM testing, since one may assume that the material prevails on the test system as supplied and that diffusion and sedimentation properties do not affect the effective in vitro dosage.

By contrast to the solids protocol variant 1, performing the EpiOcular™-EIT in according to protocol variant 2 resulted in overall high variability in tissue viability. For one dry-powder test material (TiO_2_ NM-103) it was even not possible to obtain acceptable inter-tissue variability within a total of 3 test runs. In fact, variant 2 of the EpiOcular™-EIT was performed after all dry-powder NMs tested negative in variant 1 to investigate whether the 90 min incubation period (as compared to 6 h in variant 2) and the 12 min post-exposure immersion period (as compared to 25 min in variant 2) might have been too short for effects to evolve. Thereby, this part of the study served to follow up the solids’ protocol optimization for increased sensitivity described by Kaluzhny et al*.* [65] and that has been taken up in the OECD TG 492. Taking into account the considerably higher variability observed in the EpiOcular™-EIT protocol variant 2 as compared to variant 1, it remains a matter for further investigation to address whether the increased incubation or post-exposure immersion periods are possibly disadvantageous for the testing of NMs. Generally, the updated (i.e. ‘variant 2’) test protocol of the EpiOcular™-EIT has been validated to provide excellent sensitivity [4, 65].

In the BCOP assay, solid test materials are generally applied diluted in highly deionized water. The lack of protein addition to the suspension is known to promote agglomeration [37, 66, 67], and, apart from SiO_2_ NM-203 that prevailed in small agglomerates below 100 nm diameter, all dry-powder NMs strongly agglomerated in water. If the NMs agglomerate, sedimentation prevails over diffusion, which increases the likelihood that a higher proportion of the test materials will reach the in vitro test system [63, 64]. In further addressing possible adaptations of the BCOP assay protocol for NM testing, it might be assessed whether neat testing might better mimic the in vivo situation. (In the present study, ZnO NM-111 and MWCNT NM-401 were applied as undiluted solids in the BCOP assay since homogenous mixtures in water could not be prepared for these 2 test materials.) Similarly, taking into account that tear fluid is protein-rich [68], future research might aim at addressing if a higher dispersibility of NMs by preparing test material suspensions in protein-containing media changes the outcome of the BCOP assay.

## Conclusion

All dry-powder NMs were consistently assessed as lacking eye irritation potential in both the in vitro EpiOcular™–EIT and the BCOP assay. This outcome of the present study is supported by available in vivo eye irritation data (noting that for most test materials in vivo data were only available from nanosized or bulk materials of the same composition). Nevertheless, due to the scientific limitations of the in vivo eye irritation test, also discordant outcomes between the in vivo rabbit eye irritation test and the in vitro testing strategy would not necessarily preclude the applicability of the EpiOcular™-EIT or BCOP assay.

Only two suspended NMs (Ag NM-300 K and aSiO_2_-susp), were tested in the present study, and Ag NM-300 K revealed eye irritation potential in Tier 1 (EpiOcular™-EIT). It was not possible to determine whether this material preparation has a potential for serious or irreversible eye irritation potential in Tier 2 (BCOP assay), but upon histopathological evaluation dark-brown patches were observed on the surface of the treated corneas. The dispersant of Ag NM-300 K alone (that does not contain silver nanoparticles) yielded inconclusive results. In the in vivo studies recorded in the REACH dossier for silver, dry-powder Ag NMs did not produce ocular effects, whereas Ag NMs suspended in an aqueous solution elicited minimal and fully reversible conjunctival redness in single animals only. Persistent corneal discolouration, which would lead to substance classification as ‘serious eye damage, was also not recorded in these in vivo studies.

Based upon the outcome of the present study, the 2-tier EpiOcular™ – BCOP eye irritation testing strategy appears promising to identify ‘Non-category’ dry-powder NMs, whereas a final conclusion on its applicability for NMs provided as suspensions is limited by the small number of suspended NMs tested. Of the three suspended test materials (2 of which being NMs), aSiO_2_-susp was non-irritating in both in vitro tests; Ag NM-300 K was assessed as ‘Category 1 or 2’ in the EpiOcular™-EIT and as ‘un-evaluable’ in the BCOP assay; Ag NM-300 K DIS was assessed as ‘non-Category 1 or 2’ in the EpiOcular™-EIT and as borderline positive (and ‘inconclusive’ due to high inter-assay variability) in the BCOP assay. These test results highlight that further investigations are necessary to develop a BCOP test protocol that is suitable for the evaluation of suspended NMs. Only once such an adapted test protocol is available, applicability of the BCOP assay within a 2-tier (bottom-up or top-down) testing strategy as full replacement of the in vivo eye irritation test can be substantiated. However, this goal is further impeded by the fact that reliable in vivo eye irritation data for a comprehensive set of NMs that would permit direct in vitro-in vivo comparisons of NM eye irritation potential are unavailable. Of note, also for dry-powder NMs, due to the overall low or lacking in vitro or in vivo eye irritation potential of the applied test materials, a conclusion on the applicability of the 2-tier testing strategy to differentiate between ‘Category 1’ and ‘Category 2’ NMs is not possible.

The selected panel of OECD representative NMs and organic pigments did not cover the spectrum of possible in vivo (or in vitro) eye irritating effects. To allow for a comprehensive evaluation of the 2-tier EpiOcular™ – BCOP eye irritation testing strategy, so-called benchmark NMs are required, i.e. NMs which have been tested and evaluated according to standard criteria and to which new materials may reliably be compared [57, 69]. Accordingly, comparative assessment of benchmark NMs with known in vivo ‘Category 1’ or ‘Category 2’ eye irritating effects – provided that NMs were determined that possess such effects – would allow assessing the predictivity of the two in vitro test methods, i.e. the EpiOcular™-EIT and BCOP assay for NM eye irritation testing. Keeping this limitation in mind, the outcome of the present study does indicate that the tested dry-powder representative OECD NMs do not have an eye irritation potential.

## Additional file

Additional file 1: Supplementary Information and Supplementary Information Tables to: Eye irritation testing of nanomaterials using the EpiOcular™ eye irritation test and the Bovine Corneal Opacity and Permeability assay. (DOCX 32 kb)

## Abbreviations

AC: Acceptance criteria
AUC: Analytical ultracentrifugation
BCOP: Bovine corneal opacity and permeability
BET: (Method of) Brunauer-Emmett-Teller
CAS: Chemical abstracts service
CCVD: Catalytic chemical vapour deposition
CLP: Classification, labelling and packaging
DLS: Dynamic light scattering
DMEM: Dulbecco‘s modified eagle’s medium
EpiOcular™-EIT: EpiOcular™ eye irritation test
GHS: Globally harmonised system
GLP: Good laboratory practice
HBSS: Hanks’ balanced salt solution
HE: Hematoxylin and Eosin
HSI: Histopathological score of irritation
ITV%: Relative inter-tissue variability
IVIS: In vitro irritancy score
LD: Laser diffraction
MEM: Minimum essential medium
MPS: Mean particle size
MTT: 3-[4,5-dimethylthiazol-2-yl]-2,5-diphenyltetrazolium bromide
MWCNT: Multi-walled carbon nanotube
NC: Negative control
NM: Nanomaterial
OECD: Organisation for Economic Co-operation and Development
PBS: Phosphate buffered saline
PC: Positive control
PPS: Primary particle size
REACH: Registration, evaluation, authorisation, and restriction of chemicals
SCCS: Scientific committee on consumer safety
SD: Standard deviation
SSA: Specific surface area
TEM: Transmission electron microscopy
TG: Test guideline
UN: United Nations
WPMN: Working party on manufactured nanomaterials
XPS: X-ray photoelectron spectroscopy
XRD: X-ray diffraction
