# Supplementary material for: Eye irritation testing of nanomaterials using the EpiOcular™ eye irritation test and the bovine corneal opacity and permeability assay
Source: Part Fibre Toxicol. 2016 Apr 15;13:18. doi: 10.1186/s12989-016-0128-6 (PMC4833924; doi:10.1186/s12989-016-0128-6)
Supplement: Additional file 1: — Supplementary Information and Supplementary Information Tables to: Eye irritation testing of nanomaterials using the EpiOcular™ eye irritation test and the Bovine Corneal Opacity and Permeability assay. (DOCX 32 kb) [file 12989_2016_128_MOESM1_ESM.docx]

**Supplementary Information and Supplementary Information Tables to:**

**Eye irritation testing of nanomaterials using the EpiOcular^TM^ eye irritation test and the Bovine Corneal Opacity and Permeability assay**

**Results of the EpiOcular^TM^-EIT; fulfilment of acceptance criteria**

The ACs for the NC and the PC were always met in the EpiOcular^TM^-EIT performed in accordance with either the protocol variants 1 and 2 (Additional file: Table SI-1).

AC for tissue variability: Of 17 test runs conducted in accordance to variant 1, only one single test run, the AC for tissue variability was not met, i.e. for **CeO_2_ NM-211**. However, since the calculated ITV% of 22% was only slightly higher than the 20%-threshold value and all other AC were met and since the individual values recorded for CeO_2_ NM-211 were unambiguous in respect to test substance negativity, the test was considered valid despite this deviation and was not repeated (Table 3).

Of 21 test runs for the OECD representative NMs conducted in accordance to variant 2, ITV% values exceeded 20% for 9 test runs (or laid very close to this value) with one ITV% value amounting to 73%. Overall, test runs were repeated for 6 out of 14 substances (Table 4). For the other test substances (aSiO_2_-susp, quartz dust DQ12, the three organic pigments, and talc), only the test run for aSiO_2_-susp had to be repeated because the ITV% value exceeded 20%.

- For **TiO_2_ NM-102** and **NM-105**, the ITV% values (20% and 23%, respectively) laid close to the threshold value of 20%. Since the individual measurements clearly indicated lack of toxicity, these test runs were not repeated.
- For **TiO_2_ NM-103**, the ITV% values by far exceeded the threshold value in three separate test runs, and further testing was terminated assigning the outcome of the assessment of TiO_2_ NM-103 as ‘inconclusive’.
- For both **ZnO NM-110** and **NM-111**, high ITV% values were calculated in the first test run. Therefore, both ZnO NMs were submitted to 2^nd^ test runs yielding satisfactory ITV% values.
- For **SiO_2_ NM-200** and **NM-203** and **CeO_2_ NM-211**, very high optical density values were recorded in one measurement of the respective 1^st^ test runs. Therefore, these three test substances were also submitted to 2^nd^ test runs. Here, ITV% values >20% were recorded for both SiO_2_ NMs (but not for CeO_2_ NM-211). Since all individual measurements of both the 1^st^ and the 2^nd^ test runs for SiO_2_ NM-200 and NM-203 clearly indicated lack of toxicity, their assessment was terminated after the 2^nd^ test run (Table 4).

For **aSiO_2_-susp**, the ITV% was 24% in the 1^st^ test run, whereas the ITV% of 18% calculated for the 2^nd^ test run ranged below the threshold value (Table 5).

**BCOP assay; fulfilment of the acceptance criteria**

The AC for the NC was always met. Due to high opacity scores, one PC did not meet the AC for the PC (Additional file: Table SI-2). However, since all other ACs were met and the test substance results were unambiguous, the study was assessed as being valid. For **ZnO NM-110**, **Ag NM-300K** and **Ag NM-300K DIS**, the AC for the treatment groups was not met in the 1^st^ test runs. Therefore, these substances were submitted to a 2^nd^ test run.

**Table SI-1: Mean tissue viability of the negative and positive controls applied in the EpiOcular^TM^ MTT eye irritation assay to test 16 OECD reference nanomaterials, the Ag dispersant, aSiO_2_-susp, three organic pigments, quartz dust DQ12, and talc**

|  | **Protocol for solids ‘variant 1’** | | | **Protocol for solids ‘variant 2‘** | | | | | |
| --- | --- | --- | --- | --- | --- | --- | --- | --- | --- |
| **Test substance** | **Run 1** | **Run 2** | **Run 3** | **Run 1** | **Run 2** | **Run 3** | **Run 4** | **Run 5** | **Run 6** |
| **Mean OD_570_ of NC** | 1.411 | 1.241 | 1.552 | 1.680 | 1.760 | 1.700 | 1.726 | 1.497 | 1.793 |
| **Mean NC% (and ITV%)** | 100  (7) | 100  (3) | 100  (2) | 100  (4) | 100  (2) | 100  (1) | 100  (6) | 100  (3) | 100  (7) |
| **Mean relative PC**  **(and ITV%)** | 21  (11) | 18  (5) | 24  (5) | 25  (4) | 26  (2) | 29  (7) | 23  (2) | 22  (4) | 24  (1) |

Footnote to Table SI-1:

Highly de-ionized water was used as NC and methyl acetate as PC.

**In the tests using the protocol for solids ‘variant 1’:**

- Run 1 included the testing of TiO_2_ NM-100, NM-101, NM-102, NM-103, and NM-104; ZnO NM-110; SiO_2_ NM-203; CeO_2_ NM-211 and NM-212.
- Run 2 included the testing of TiO_2_ NM-105; ZnO NM-111; SiO_2_ NM-200; MWCNT NM-400, NM-401, NM-402.
- Run 3 included the testing of Ag NM-300K and Ag NM-300K DIS.

**In the tests using the protocol for solids ‘variant 2’:**

- Run 1 included the tests of TiO_2_ NM-100, NM-101, NM-102, NM-103, NM-104, and NM-105; ZnO NM-110 and NM-111; SiO_2_ NM-200 and NM-203; CeO_2_ NM-211 and NM-212; MWCNT NM-400 and NM-401
- Run 2 included the 2^nd^ testing of TiO_2_ NM-103; ZnO NM-110 and NM-111; SiO_2_ NM-200 and NM-203; CeO_2_ NM-211
- Run 3 included the 3^rd^ testing of TiO_2_ NM-103
- Run 4 included the testing of the three organic pigments and talc
- Run 5 included the 1^st^ testing of aSiO_2_-susp
- Run 6 included the 2^nd^ testing of aSiO_2_-susp.

The mean tissue viability of 2 tissues / test group is expressed relative to the corresponding negative control value further indicating (in brackets) the ITV%

Abbreviations: ITV%: Relative inter-tissue variability; NC: Negative control; OD: Optical density; PC: Positive control

**Table SI-2: Mean opacity and permeability values and corresponding *in vitro* irritation score (IVIS) of the negative and positive controls used to test 16 OECD reference nanomaterials, the Ag NM dispersant, aSiO_2_-susp, three organic pigments, quartz dust DQ12, and talc in the BCOP assay**

|  | |  |  | |  |  |  |  |  |  |  |
| --- | --- | --- | --- | --- | --- | --- | --- | --- | --- | --- | --- |
| **Test substance** | **Parameter** | | | **Run 1** | **Run 2 ^a^** | **Run 3 ^b^** | **Run 4** | **Run 5** | **Run 6** | **Run 7** | **Run 8** |
| **NC** | **Mean opacity value ± SD** | | | 4.7 ± 5.7 | 1.6 ± 2.0 | 9.1 ± 6.0 | 3.8 ± 2.5 | 1.4 ± 2.8 | -1.1 ± 1.1 | 8.1 ± 4.9 | 7.3 ± 3.3 |
|  | **Mean permeability value ± SD** | | | 0.01 ± 0.02 | 0.04 ± 0.03 | 0.02 ± 0.01 | 0.00 ± 0.00 | -1.10 ± 1.10 | 0.04 ± 0.05 | 0.01 ± 0.00 | 0.00 ± 0.00 |
|  | **IVIS ± SD** | | | 4.9 ± 5.7 | 2.2 ± 1.6 | 9.5 ± 5.9 | 3.9 ± 2.5 | 0.0 ± 0.0 | -0.5 ± 1.1 | 8.2 ± 4.9 | 7.3 ± 3.3 |
| **PC** | **Mean opacity value ± SD** | | | 78.5 ± 16.4 | 81.9 ± 7.5 | 67.8 ± 9.4 | 170.6 ± 8.8 | 123.6 ± 5.3 | 133.1 ± 23.2 | 83.0 ± 11.1 | 70.2 ± 25.4 |
|  | **Mean permeability value ± SD** | | | 3.0 ± 0.8 | 4.4 ± 0.5 | 3.4 ± 0.9 | 2.9 ± 1.1 | 3.1 ± 0.3 | 3.0 ± 0.4 | 1.3 ± 0.2 | 3.0 ± 0.5 |
|  | **IVIS ± SD** | | | 123.0 ± 17.4 | 147.3 ± 9.0 | 118.7 ± 8.8 | 214.5 ± 15.9 ^c^ | 170.7 ± 1.5 | 178.0 ± 21.3 | 102.4 ± 10.1 | 115.1 ± 33.3 |

Footnote to Table SI-2:

Highly de-ionized water was used as NC. For Ag NM-300K and its dispersant, Ag NM-300K DIS, that were supplied as suspensions, 1% (w/v) sodium hydroxide was used as PC. For all other test substances, 20% (w/v) imidazole dissolved in highly de-ionized water was used as PC. Ag NM-300K and Ag NM-300K DIS were tested using the BCOP treatment protocol for liquids (10 min test substance exposure, 2 hours post-exposure incubation) while all others were tested according to the treatment protocol for solids (4 hours test substance exposure without further post-incubation).

- Run 1 included the testing of TiO_2_ NM-100 and MWCNT NM-400, NM-401, NM-402.
- Run 2 included the testing of TiO_2_ NM-101, NM-103, and NM-104; and ZnO NM-110 (1^st^ testing)
- Run 3 included the testing of TiO_2_ NM-102; ZnO NM-111; SiO_2_ NM-200 and NM-203; CeO_2_ NM-211 and NM-212.
- Run 4 included the testing of TiO_2_ NM-105 and ZnO NM-110 (2^nd^ testing).
- Run 5 included the 1^st^ testing of Ag NM-300K and Ag NM-300K DIS.
- Run 6 included the 2^nd^ testing of Ag NM-300K and Ag NM-300K DIS
- Run 7 included the testing of Pigment Yellow 95, Pigment Black 32, quartz dust DQ12, and talc
- Run 8 included the testing of Pigment Red 57:1 and aSiO_2_-susp.

a: Two corneas could be evaluated for the NC, only, due to leakage of one corneal holder during fluorescein incubation.

b: The opacity values of the test substance and PC are presented without subtraction of the relatively high NC-opacity value.

c: Due to high opacity scores, the IVIS of the PC did not meet the corresponding acceptance criterion (i.e. it lay outside the historical mean in-house control value ± 2x the standard deviation (i.e. <88.0 or >147.1). However, since all other acceptance criteria were met and the test substance results were unambiguous, the study was assessed as nevertheless being valid.
